# Computational screening of known broad-spectrum antiviral small organic molecules for potential influenza HA stem inhibitors

**DOI:** 10.1371/journal.pone.0203148

**Published:** 2018-09-04

**Authors:** Shilu Mathew, Asmaa A. Al Thani, Hadi M. Yassine

**Affiliations:** 1 Biomedical Research Center, Qatar University, Doha, Qatar; 2 College of Health Sciences, Qatar University, Doha, Qatar; UMR-S1134, INSERM, Université Paris Diderot, INTS, FRANCE

## Abstract

**Background:**

With the emergence of new influenza virus strains that are resistant to current inhibitors such as oseltamivir (anti-neuraminidase (NA)) and amantadine (anti-M2 proton channel), influenza A viruses continue to be a serious threat to the public health worldwide. With this in view, there is a persistent need for the development of broader and more effective vaccines and therapeutics. Identification of broadly neutralizing antibodies (bNAbs) that recognize relatively invariant structures ‎on influenza haemagglutinin (HA) stem has invigorated efforts to develop universal influenza vaccines.

**Aim:**

The current computational study is designed to identify potential flavonoid inhibitors that bind to the contact epitopes of HA stem that are targeted by broadly neutralizing antibodies (bNAb).

**Method:**

In this study, we utilized the three-dimensional crystallographic structure of different HA subtypes (H1, H2, H5, H3, and H7) in complex with bNAb to screen for potential broadly reactive influenza inhibitors. We performed Quantitative Structure-Activity and Relationship (QSAR) for 100 natural compounds known for their antiviral activity and performed molecular docking using AutoDock 4.2 suite. Furthermore, we conducted virtual screening of 1413 bioassay hit compounds by using virtual lab bench CLC Drug Discovery.

**Results:**

The results showed 18 lead flavonoids with strong binding abilities to bNAb epitopes of various HA subtypes. These 18 broadly reactive compounds exhibited significant interactions with an average of seven Hbonds, docking energy of -22.43 kcal·mol^−1^, and minimum interaction ‎ energy of -4.65 kcal·mol^−1^, with functional contact residues. Procyanidin depicted strong interactions with group 1 HAs, whereas both sorbitol and procyanidin exhibited significant interactions with group 2 HAs.

**Conclusion:**

Using in silico docking analysis, we identified 18 bioactive flavonoids with potential strong binding cababilities to influenza HA-stems of various subtypes, which are the target for bNAb. The virtual screened bioassay hit compounds depicted a high number of Hbonds but low interaction and docking values compared to antiviral flavonoids. Using structure-based design and nanotechnology-based approaches, identified molecules could be modified to generate next generation anti-influenza drugs.

## Background

Influenza A viruses (IAV) are a major cause of respiratory illness and result in significant mortality and morbidity worldwide [1]. Although influenza vaccine has been available since 1938, the vaccine content has to be assessed every year due to the continual change in hemagglutinin protein (HA), which is the main target for neutralizing antibodies [2]. In addition to vaccines, there are few drugs that are used to treat severe influenza infections [3]. Neuraminidase and adamantanes inhibitors are two classes of anti-IAV drugs, approved to treat IAV infections by targeting the viral components: Matrix proton channel (M2) and Neuraminidase glycoprotein (NA) [2, 4]. However, H1N1 and H3N2 strains resistance to both drugs, especially to amantadine, are prevalent worldwide [5]. In fact, up to 100% of the H1N1 subtypes were recorded resistant to oseltamivir as of 2009 [5]. In 2009, the swine-origin pandemic H1N1 (pH1N1) replaced the pre-2009 oseltamivir-resistant H1N1 variants and has now become one of the dominant circulating seasonal influenza virus strains [5, 6]. Neuraminidase inhibitors are still effective in treating circulating strains including adamantine-resistant one. Nonetheless, resistant strains might sporadically emerge and spread worldwide considering the wide use of inhibitor drugs and the unfortunate poor vaccine efficacy [7].

Broadly neutralizing antibodies (bNAb) that target conserved epitopes on HA stem have been frequently isolated from phage-display libraries, plasma cells of immunized mice, or memory B cells of immune donors [8]. Some of these antibodies are IAV group specific such as CR6261 [9], F10 [10], and CR8020 [11], or with broader activity that target almost all IAV strains such as CR9114 [12], FI6 [13], and many others [14, 15], such discoveries has opened new doors toward passive immunotherapy and development of novel influenza vaccines with broader activity [16].

We have reported on the structure-based design of a stabilized H1 stem nanoparticles (H1–SS–np) that protect against heterogeneous highly pathogenic H5N1 virus in both mice and ferrets [17]. In the same study, we showed that passive transfer of immunoglobulin from H1–SS–np–immunized mice to naive mice conferred protection against H5N1 challenge, highlighting the importance of conserved key structural elements as targets for vaccination and therapeutic purposes [17]. Considering the restrictions and limitations of monoclonal antibodies (mAb) use in therapeutics due to their cost, autoimmunity potentials, and the anti-mAb immune response, these sites of vulnerability on HA-stem could be an ideal target for drug-based therapeutics in the form of small molecules [18].

Numerous approaches have been used to explore new antiviral drugs including *in silico* and *in vitro* screening of known compounds for anti-IAV activity, as well as structure-based design of new antivirals drugs against specific viral molecules [19]. Presently, several flavonoids have been identified with anti-IAV activity by inhibiting neuraminidase [20, 21]. Flavonoids possess many biochemical properties, but the best-described property of almost every group of flavonoids is their capacity to act as antioxidants [22] and hapatoprotective activities [23]. In over-all, flavonoids are exciting molecules combining an aromatic nature with numerous hydrophilic groups. These aromatic properties play a significant role in protein-ligand and protein-protein interactions [24, 25]. Further, the hydroxyl (OH) functional group (hydrophilic nature) of flavonoids enables water displacement, which is a key determinant for ligand’s affinity [26]. Some of the flavonoids that exhibit anti-IAV activity are: phytochemicals ajoene, baicalein, catechin, coumarin, menthol, theaflavin, ursolic acid, carvacrol, tinosporon, andrographolide, and cucurmin [27–29].

In this study, we utilized the complex three-dimensional structure from PDB database of various HA subtypes with bNAb to screen for strong flavonoid binders to conserved stem region. We performed Quantitative Structure-Activity and Relationship (QSAR) for 100 natural compounds known for its antiviral activity and performed molecular docking using AutoDock 4.2 suite. Furthermore, we conducted virtual screening of 1413 bioassay hit compounds by using CLC Drug Discovery Workbench 3.02.

## Methods

### Structural models of influenza A stem from Group 1 and Group 2

PDB database was used to retrieve the X-ray crystal structure of various HA subtypes representing group1 and group 2 influenza viruses. This include H1-HA structure in complex with CR6261 Fab (resolution of 2.2 Å; PDB# 3GBN;UniProtKB AC: Q9WFX3) (Figure A S1 Fig) [30], H2 structure in complex with C179 Fab (resolution of 2.9Å; PDB# 4HLZ; UniProtKB AC: C7S226) (Figure B S1 Fig) [3], H5 structure in complex F10 ScFv (resolution of 3.2Å; PDB# 3FKU; UniProtKB AC: Q6DQ34) (Figure C S1 Fig) [31], H3 structure in complex with CR8020 Fab (2.85Å of resolution; PDB ID# 3SDY; UniProtKB AC: A0A097PF39) (Figure D S1 Fig) [32] and H7 structure in complex with CR9114 Fab (resolution of 5.75; Å PDB# 4FQV; UniProtKB AC: Q6VMK1) (Figure E S1 Fig) [33]. Conserved epitopes of group 1 and group 2 HA structural models are shown in S1 Fig.

### Protein preparation

PDB structures of H1, H2, H3, H5, and H7 to be docked with flavonoids were imported in PDB format into AutoDock 4.2 and were assigned with specifically polar hydrogen for appropriate treatment of electrostatic docking. PDBQT file was used as coordinate files: includes atom types and atomic partial charges [34]. Protein preparation using HA structure of H1N1 complex for virtual screening of small molecules was performed on CLC Drug Discovery Workbench. The macromolecule (protein epitopes) were then refined with the Hbonds (HB) assignment (water orientations, at neutral pH), and energy was minimized with MMFF94 force field [35]. The structure was then refined, making a minimization of the conformational energy to generate 3D molecule structures on import [36]. Post-processing step was applied for small molecules with no rotatable bonds with the energy window of 5 kcal/mol [37]. The energy window was increased by 0.25 kcal/mol for each rotatable bond present in the molecule. A grid for the protein was generated by using site around the centroids of selected residues in both AutoDock 4.2 and CLC Drug Discovery Workbench. The contact residues on H1 stem were: HIS8, HIS28, VAL30, ASN31, LEU32, ASP70, GLY71, TRP72, THR92, GLN93, ILE96, ASN97, VAL103, ASN104, ILE107, THR100 (CR6261)[17]; on H2 stem: VAL18, ASP19, GLY20, LYS38, TRP21, THR41, PHE45, ASP46, LYS43, GLN42, HIS38, VAL52, ILE56, PRO293, THR291, LEU292, ILE42, LYS40, THR318 (C179)[3]; on H5 stem HIS38, PHE55, GLY20, HIS18, ASP19, TYR102, GLN42, ILE45, MET54, PHE55, THR41 (F10)[10]; on H3 stem GLU15, GLY16, VAL18, ASP19, LEU318, GLU325, GLN34, ALA35, ALA36, ARG25, GLY33, THR32, ASN146, GLY150, GLU30 (CR8020)[38] and H7 ILE18, ASP19, ALA36, LYS38, GLY20, TRP21, THR41, ILE45, GLN42, ASP46, THR49, SER40, LEU52, ILE56, ALA292, ARG291, VAL293, ILE45 (CR9114)[12]. Contact residues on HA stem with bNAb are depicted in S2–S6 Figs and summarized in S1 Table.

### Ligand preparation

A total of hundred antiviral bioactive flavonoids were selected for *in silico* binding screening to the bNAb epitope of the HA stem (S2 Table). The screening was done sequentially by testing the binding to H1 subtype, and those with potential binding affinities were further screened for binding to the rest of group 1 and then group 2 HA subtypes. ACD Chemsketch was used to generate 3D structures of the natural molecules known for their antiviral properties [39]. 1413 compounds were selected from PubChem database (Bioassay based) according to the biological activity against IAV with IC50 ≤ 1 nM (605 active compounds) and IC50 ≤ 1 μM (808 active compounds) for *in silico* docking analysis [40]. The 2D structures for screened compounds were obtained from PubChem [41]. Ligand preparation in CLC Drug Discovery was applied using freely available program “Balloon” which is used for the 3D structure generation [42]. MedChem Designer tool was used to sketch 3D molecule and import as SMILES strings format in CLC Drug Discovery Workbench [43].

### Quantitative structure-activity relationship (QSAR)

‎A virtual model for property evaluation of chemicals was used to analyze the QSAR of selected ligands to evaluate biological activities and physiochemical properties [44]. With the recent advances in structure-based approaches in designing new drugs and vaccines, statistically-based QSAR analysis helps in guiding optimization of generated ‎product in terms of their biological and chemical properties. QSAR models predict biological activities based on chemical structures. Mutagenicity model, physiochemical characters of ligands such as logP, carcinogenicity model, developmental toxicity model, and ready biodegradability model, were determined for the selected flavonoids.

### Docking protocol

Molecular docking studies for bioflavonoids were done by using AutoDock 4.2 MGL tools [45]. Protein structures were prepared by creating PDBQT file that contains partial charges and hydrogen atoms estimated by Gasteiger charges [46]. PDBQT file was prepared for ligand via AMBER force field by adding hydrogen atoms. The contact residues were selected from the string to generate grid parameters to target the potential binding site. Docking parameter file was generated to run rescoring for higher accuracy [47]. Finally, each binding mode was scored using the scoring function available in AutoDock and the best binding mode with the corresponding interaction energy (IE) was identified. For comparative screening analysis, 1413 bioassay hit compounds were selected to screen for their binding to H1 HA stem by creating ligand based projects for all the chemical structures downloaded in the SDF file format. CLC Drug Discovery Workbench was then used to generate ten best poses for each conformation of the best docking energy (DE) based on scoring functions. Docking wizard was used by applying default MolDock optimizer algorithm with the following docking parameters including 200 number of runs, maximum iterations 2000, crossover rate 0.90, scaling factor 0.50 and RMSD thresholds for similar cluster poses were set as 1.00 [35]. The best-ranked compounds were selected on the basis of HB and DE and were visualized by using CLC Drug Discovery Visualization tool.

## Results

### VEGA-QSAR profiling of screened products

Different biochemical properties of potential bioflavonoids were predicted by using VEGA-QSAR analysis. Results evaluated by QSAR models could be effective to determine the chemical properties of chosen compounds, thus, limiting the necessity of *in vitro* and *in vivo* evaluation for only potential compounds. More than 65% of the chosen flavonoids were non-mutagen, non-toxic, non-carcinogen and readily biodegradable. Predicted values for hundred bioflavonoids using VEGA-QSAR and their applicability domain analysis for various models have been summarized in S3 Table. The log *P* value of the chosen flavonoids ranged from -0.01 to 7.05 log units.

### Molecular interactions of flavonoids with group 1 subtypes: H1, H2, and H5

**Fig 1:**
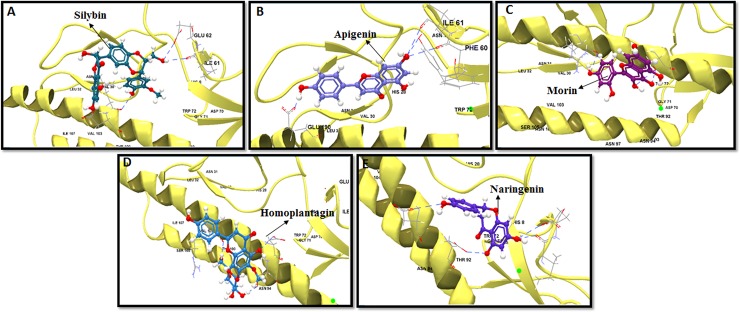
Computational docking of flavonoids on H1 stem. Top five binders are shown: (a) Silybin, (b) Apigenin, (c) Morin, (d) Homoplantagnin and (e) Naringenin. H1 stem region is depicted in ribbon with yellow color. HB interaction are denoted in dashed blue color.

**Fig 2:**
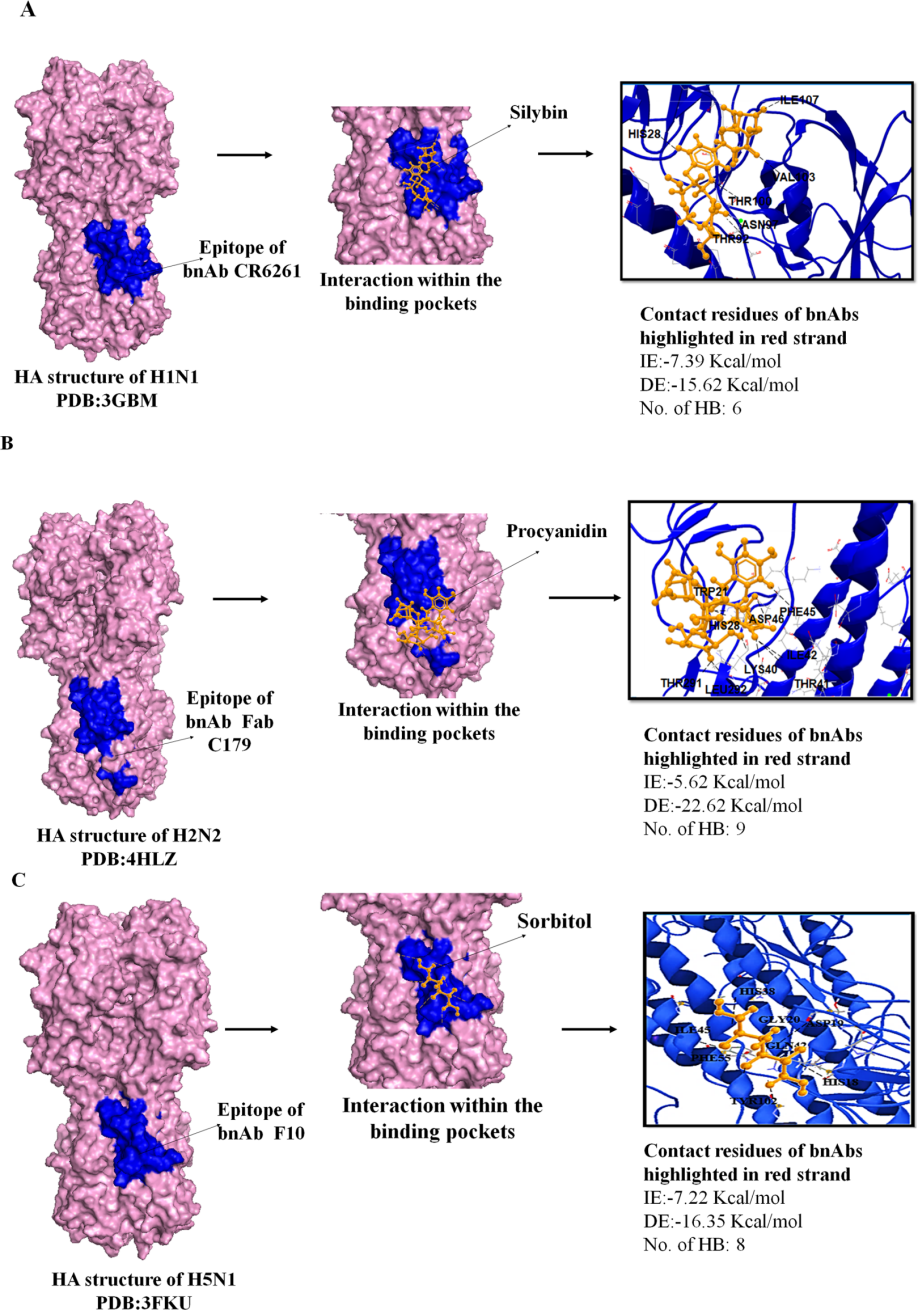
Docking of three main flavonoids (silybin, procyanidin and sorbitol) to group 1 HA-Stem (H1, H2 and H5). **Analysis was done with AutoDock 4.2 Software. Analysis was done by considering the important parameters including HB interaction, IE, and DE.** A. Left panel shows H1 HA structure in light pink with CR6261 bNAb epitope shown in dark blue. Middle panel shows interaction of silybin denoted in orange color to the bNAb epitope and right panel shows anchoring residues between silybin molecule and stem epitope of bNAb. HB interaction is shown in black color. B. Left panel shows H2 HA structure in light pink with Fab C179 bnAb epitope shown in dark blue. Middle panel shows interaction of procyanidin to the bNAb epitope and right panel shows contact residues between procyanidin molecule and stem epitope of bNAb. C. Left panel shows H5 HA structure in light pink with F10 bNAb epitope shown in dark blue. Middle panel shows interaction of sorbitol to the bNAb epitope and right panel shows contact residues between sorbitol molecule and stem epitope of bNAb.

In our *in silico* docking analysis, 100 natural bioactive flavonoids were initially docked with H1 subtype HA viral protein, from which, 46 flavonoids showed multiple HB and electrostatic interactions to the contact residues of bNAb epitope. Five best binders with a maximum number of HB interaction included silybin, apigenin, morin, homoplantagin, and naringenin. Five best conformations of best binders formed are illustrated in Fig 1. Flavonoid silybin exhibited the best atomic interaction ‎ with H1 HA-stem residues HIS28, THR 40, ILE96, TRP 72, THR92, ASN97, THR100, VAL103, and ILE107, with minimum IE of -7.36 Kcal/mol and a DE of -15.62 kcal/mol (Fig 2(A)). Apigenin and naringenin potentially formed five and three HB with the alpha helix region of the H1 stem with IE of -6.36 Kcal/mol (DE: -27.32 Kcal/mol) and -5.66 Kcal/mol (DE: -28.74 Kcal/mol), respectively (Fig 1B and 1E). Morin and homoplantagnin formed only four HB but showed high interaction with an average of -7.56 Kcal/mol IE and -17.36 Kcal/mol DE (Fig 1C and 1D). Homoplantagnin formed marginally higher DE with CR6261 epitope contact residues GLY71, THR92, ILE96, and VAL103 with an IE of -8.69 Kcal/mol, and DE of -18.65 Kcal/mol (Fig 1D). Other bioflavonoids such as amentoflavone, baicalein, scutellarin, thioflavin and matteflavoside formed highest IE with only three HB, with an average DE of -14.32 Kcal/mol (S4 Table). Minimum of four HB confirmation was extended by sorbitol, epicatechin, procyanidin, quercetin, luteolin and honokiol with an average IE of -5.64 Kcal/mol and DE ranging between -5.63 Kcal/mol and -18.36 Kcal/mol. Most of the screened bioflavonoids interacted repeatedly with H1 stem fusion peptide residues ASN104 (helix region), and THR100 (loop region). Additionally, these bioflavonoids also anchored several linear and discontinuous residues located on the H1 HA-stem. The HB formed between each flavonoid compound and the H1 HA-stem with its DE, IE, and number of residue interaction with labels are denoted in S4 Table.

**Fig 3:**
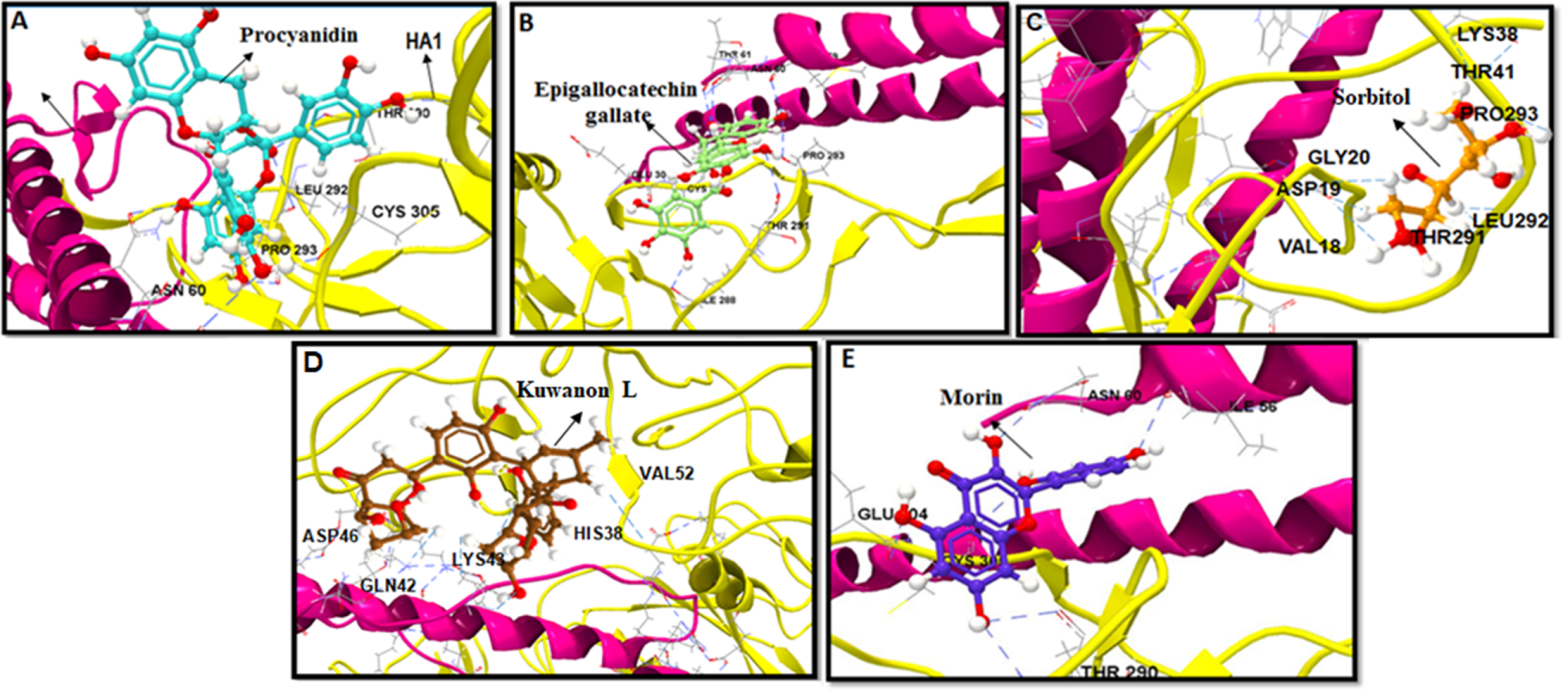
Computational docking of flavonoids on H2 stem. Top five binders are shown: (a) Procyanidin, (b) EGCG, (c) Sorbitol, (d) Kuwanon L and (e) Morin. H2 stem region is depicted in ribbon with HA1 in pink and HA2 in yellow color. HB interaction are denoted in dashed blue color.

Out of the forty-six bioflavonoid components that showed potential significant binding to H1 HA-stem, only twenty-three compounds interacted with a minimum of three HB with H2 and H5 subtypes (S5 Table). The best five binders with H2 stem subtype included procyanidin, Epigallocatechin gallate (EGCG), sorbitol, kuwanon L and morin, which fitted well in the defined active pockets of the fusion peptide with a minimum IE values of -5.62 kcal·mol^−1^, -5.98 kcal·mol^−1^, -7.54 kcal·mol^−1^ and -5.85kcal·mol^−1^, respectively and with average DE of -20.45 kcal·mol^−1^ (Fig 3). Procyanidin formed highest of nine HB interaction with binding residues TRP21, THR41, PHE45, ASP46, THR291, LEU292, ILE42, LYS40, and THR318 of the H2 stem in the beta strand (Fig 2(B)). EGCG, morin, and sorbitol also formed maximum number of eight HB and with an average DE of -19.67 kcal·mol^−1^ (Fig 3B, 3C and 3E). Scutellarin, spirooligannone, salicin and 7-O-galloytricetiflavone exhibited six HB interaction at the C179 binding pocket with a minimum average IE greater than -7.22 kcal·mol^−1^. Minimum of three to five HB were made by hesperidin, glycyrrhiza flavonol A, silybin, isorhamnetin, baicalein, quercetin, luteolin, honokiol, apigenin, isoliquiritigenin and salicylic acid, with DE between -8.41 kcal·mol^−1^ to -16.36 kcal·mol^−1^ (S5 Table). Interactions of the above flavonoids were also located at beta strand (TRP21 to VAL52), beta-bridge (VAL18-GLY20), loop subdomains and bend in distal HA region (ILE56, THR291-PRO293 and THR318) in the topological domain (S5 Table). Most of the HB interactions from the 23 compounds were observed in the N-terminus of the HA2 subunit, helix A, and beta strand, within the hydrophobic pocket incorporating residues VAL18, ASP19, GLY20, TRP21, LYS38, THR41, GLN42, PHE45 and VAL 52 (S5 Table).

**Fig 4:**
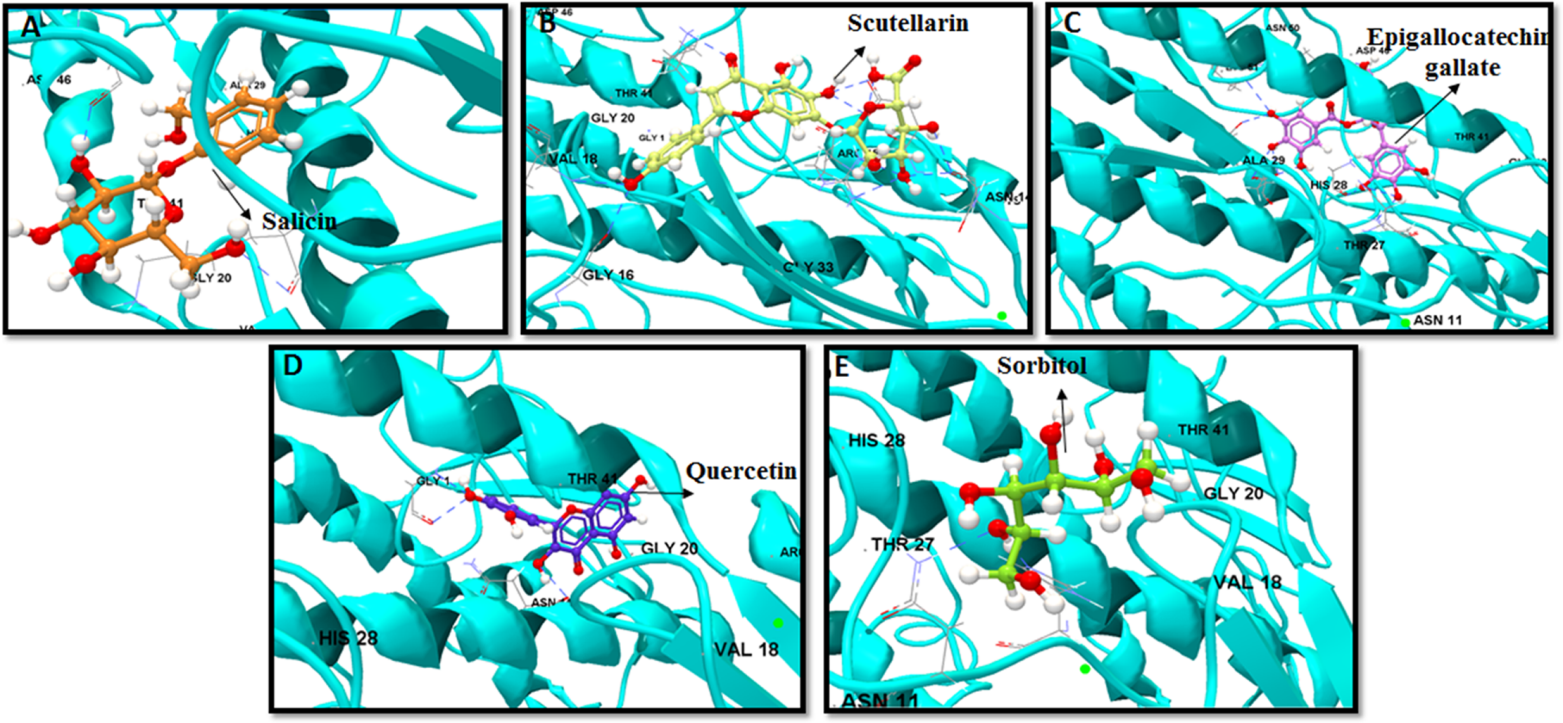
Computational docking study of flavonoids on H5 stem. Top five binders are shown: (a) Salicin, (b) Scutellarin, (c) EGCG, (d) Quercetin and (e) Sorbitol. H5 stem region is depicted in ribbon with HA1 and HA2 in blue color. HB interaction are represented in dashed blue color.

We next evaluated the top five binders interaction with H5 stem, epitope of the F10 bNAb. salicin, scutellarin, EGCG, quercetin and sorbitol extended an average of seven HB with DE of -11.34 kcal·mol^−1^ (Fig 4). Among the five best binders, only sorbitol formed eight HB with residues HIS38, PHE55, GLY20, HIS18, ASP19, TYR102, GLN42 and ILE45 with IE of -7.22kcal·mol^−1^ and DE of -12.45 kcal·mol^−1^ (Fig 2(C)). EGCG was the second best binder, extended seven HB interaction each with residues GLY20, HIS18, ASP19, TYR102, GLN42, ILE45, and MET54 of the H5 stem and with IE of -7.36 kcal·mol^−1^ and DE of 16.35 kcal·mol^−1^ (Fig 4(C)). Salicin, scutellarin and quercetin formed an equal number of six HB and with IE greater than -7.93 kcal·mol^−1^ (Fig 4A, 4B and 4D). Residues at positions HIS18, ASP19, GLY20, HIS38, and PHE55 within H5 stem, formed frequent interaction with all five best compounds. The hydrophobic residues MET54 and PHE55 of the beta strand, which lies in main-chain of the fusion peptide formed HB with scutellarin, silybin, glycyrrhiza flavonol A, quercetin, apigenin, isoliquiritigenin, and salicin (S5 Table). Apart from the best binders, procyanidin, morin, scutellarin, silybin, isorhamnetin, quercetin, salicin, and amentoflavone formed six HB with average IE and DE of -4.37 kcal·mol^−1^ and -10.46 kcal·mol^−1^, respectively (S5 Table).

### Molecular interactions with group 2 subtypes H3, and H7

**Fig 5:**
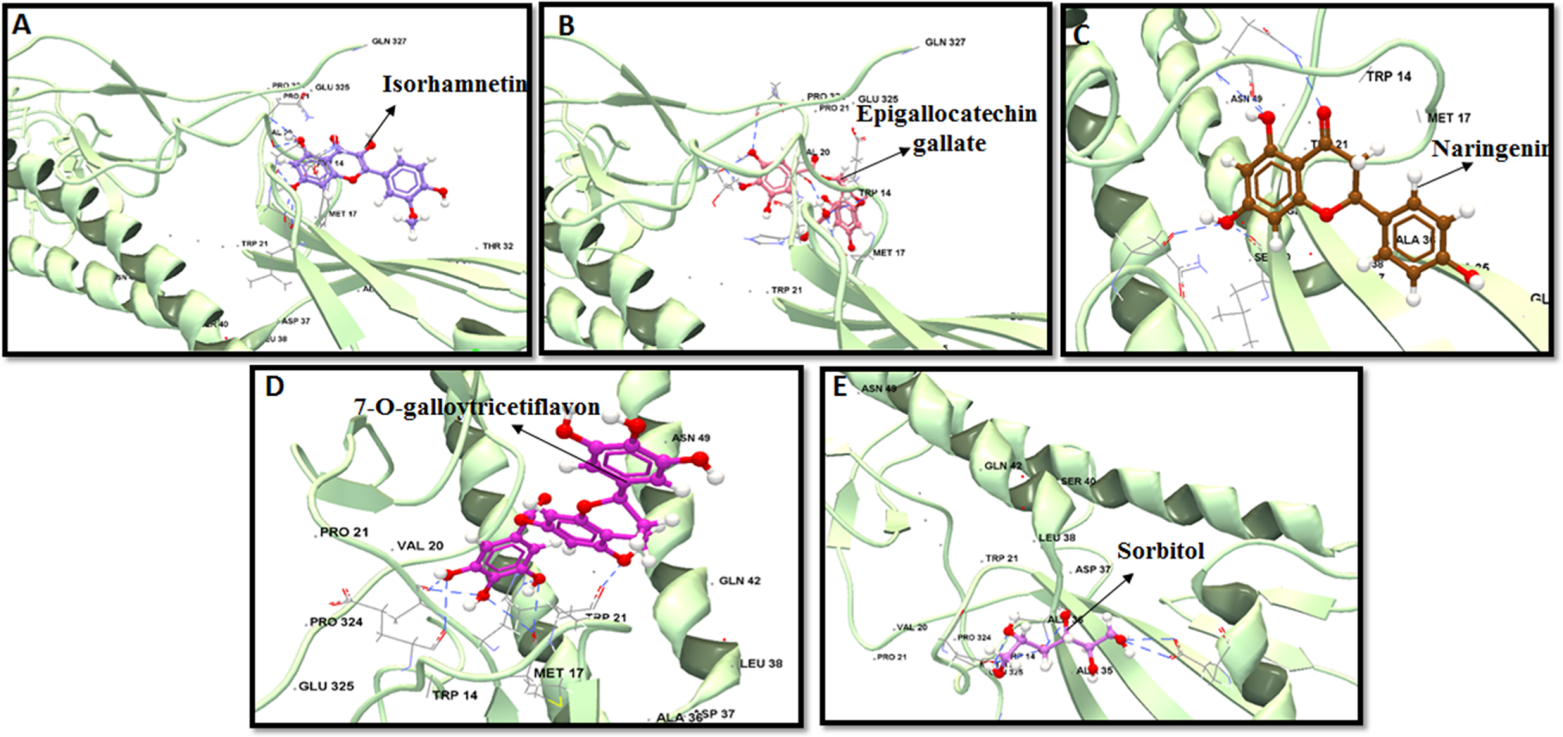
Computational docking study of flavonoids on H3 stem. Top five binders are shown: (a) Isorhamnetin, (b) EGCG, (c) Naringenin, (d) 7-O-galloytricetiflavon and (e) Sorbitol. H3 stem region is depicted in ribbon with HA1 and HA2 in shaded green color. HB interaction are represented in dashed blue color.

**Fig 6:**
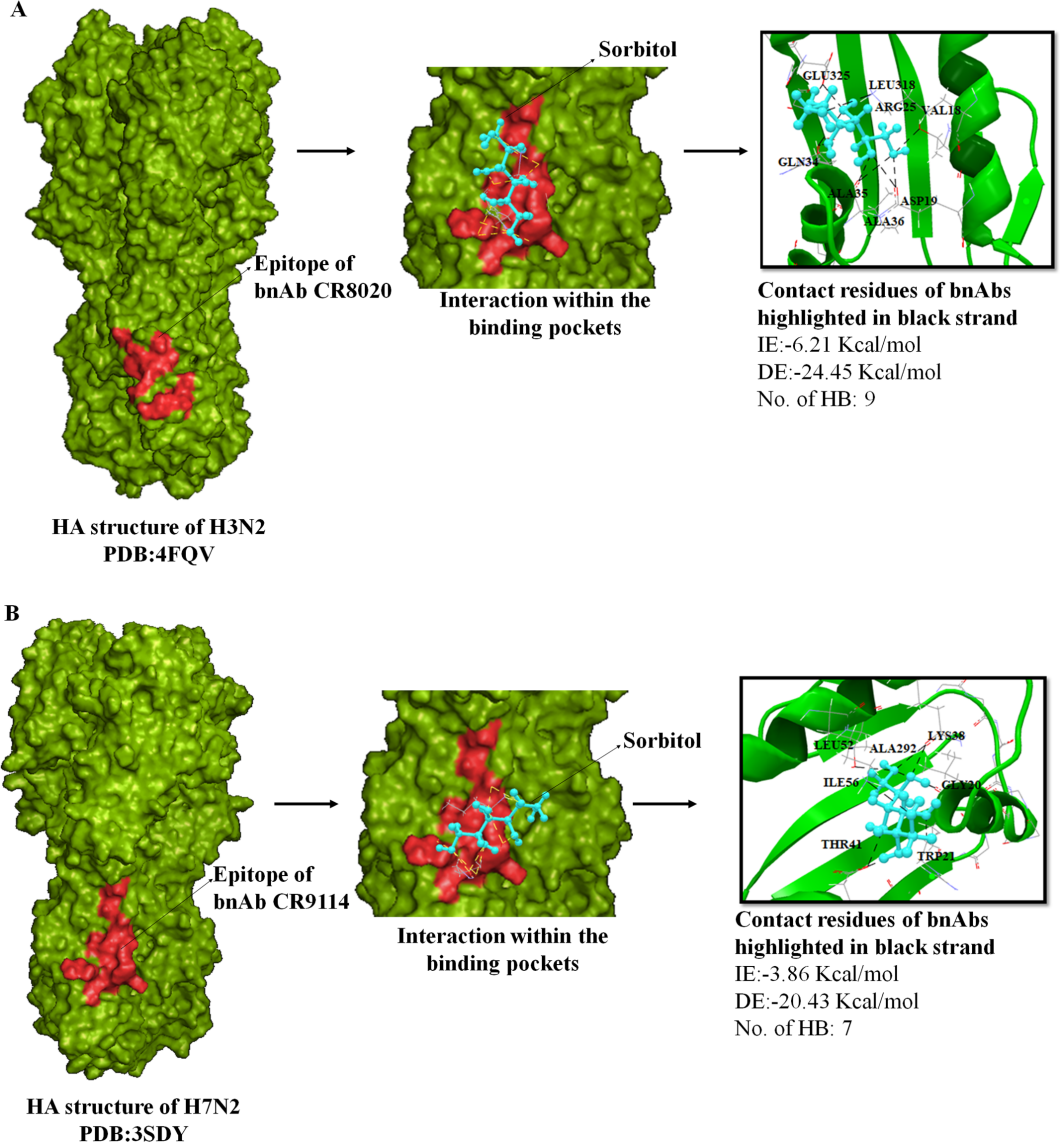
Docking of flavonoids sorbitol to group 2 HA structures, H3 and H7. **Analysis was done with AutoDock 4.2 Software. Analysis was done by considering the important parameters including HB interaction, IE, and DE.** A. Left panel shows H3 HA structure in light green with CR8020 bNAb epitope shown in red. Middle panel shows interaction of sorbitol denoted to the bNAb epitope and right panel shows anchoring residues between sorbitol molecule and stem epitope of bNAb. HB interaction is shown in black color. B. Left panel shows H7 HA structure in green with CR9114 bNAb epitope in red. Middle panel shows interaction of sorbitol to the bNAb epitope and right panel shows contact residues between sorbitol molecule and stem epitope of bNAb.

Out of 23 bioflavonoid molecules that exhibited potential significant interaction with group1 HA-stems, only 18 flavonoids docked with more than four HB interactions and a minimum energy to the H3 stem. H3 stem best binders consist of isorhamnetin, EGCG, naringenin, 7-O-galloytricetiflavone and sorbitol (Fig 5). Both sorbitol (IE:-6.21 kcal·mol^−1^ and DE:-24.45 kcal·mol^−1^) and procyanidin (IE:-5.62 kcal·mol^−1^ and DE:-13.45 kcal·mol^−1^) extended a maximum of nine HB (S6 Table). Sorbitol bound with the same of bNAb CR9114 stem epitope residues VAL18, ASP19, LEU318, GLU325, GLN34, ALA35, ALA36, ARG25, GLY33 (Fig 6A), while procyanidin bound with residues GLN34, ALA35, ALA36, ARG25, GLY33, THR32, ASN146, GLY150, and GLU30 of the H3-stem epitope (S6 Table). Almost five HB were extended by morin (IE:-6.66 kcal·mol^−1^, DE:-16.31 kcal·mol^−1^), apigenin (IE:-3.24 kcal·mol^−1^, DE:-11.45) and kuwanon L (IE:—3.32 kcal·mol^−1^, DE:-9.99 kcal·mol^−1^), in the alpha helix A region within the hydrophobic pocket (S6 Table). Maximum number of HB were observed in the outermost strand (HA2 residues GLU30–ALA36) of the 5-stranded β-sheet and the HA2 residues GLU15–ASP19 in the C-terminus portion of the fusion peptide. Of the 15 residues that constituted the contact epitopes of CR8020 bNAb, only residue ASP19 was in common interaction with most of the bioflavonoids including morin, scutellarin, apigenin, kuwanon L, baicalein, naringenin, isoliquiritigenin, salicin, 7-O-galloytricetiflavone, sorbitol, luteolin and amentoflavone (S6 Table).

**Fig 7:**
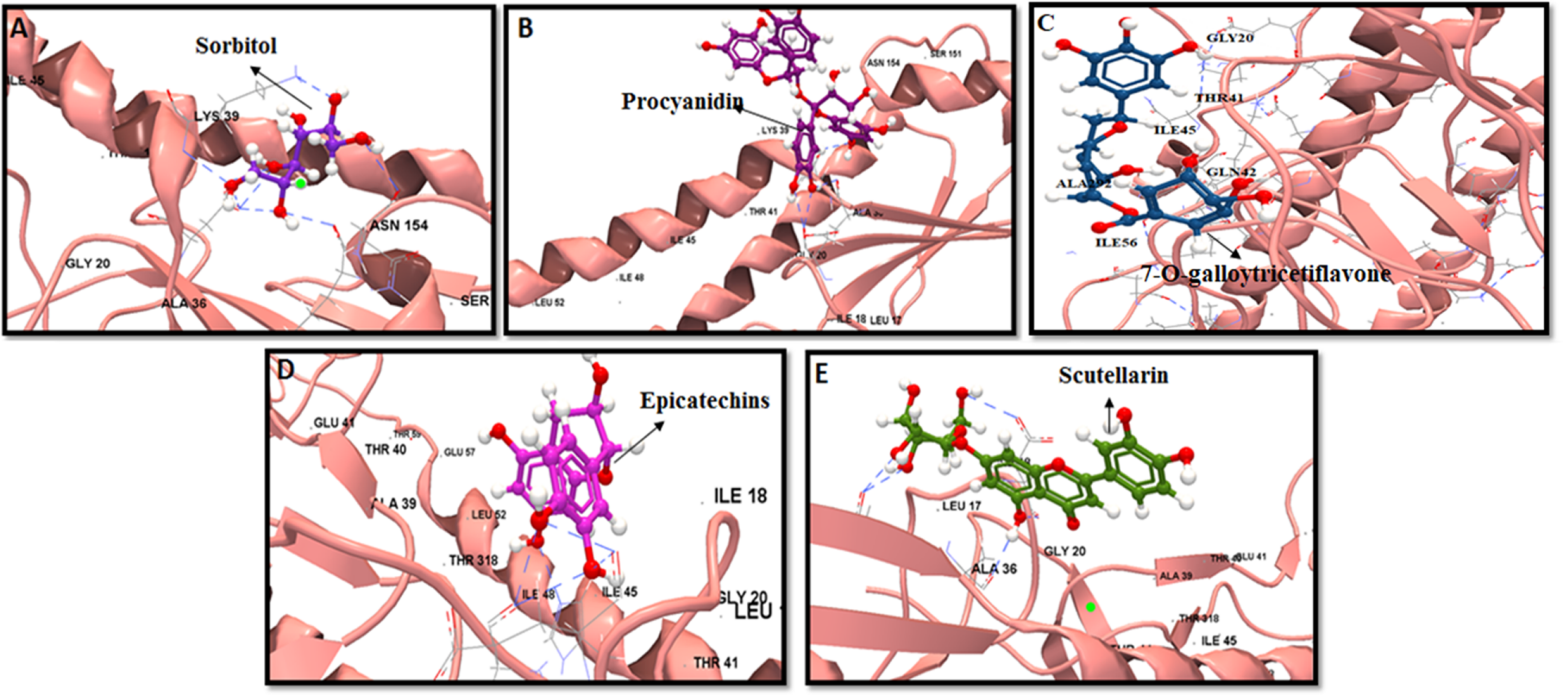
Computational docking of flavonoids on H7 stem. Top five binders are ‎shown: (a) Sorbitol, (b) Procyanidin, (c) 7-O-galloytricetiflavon, (d) Epicatechins and (e) Scutellarin. H7 stem region is depicted in ribbon with HA1 and HA2 in shaded pink color. HB interaction are represented in dashed blue color.

**Fig 8:**
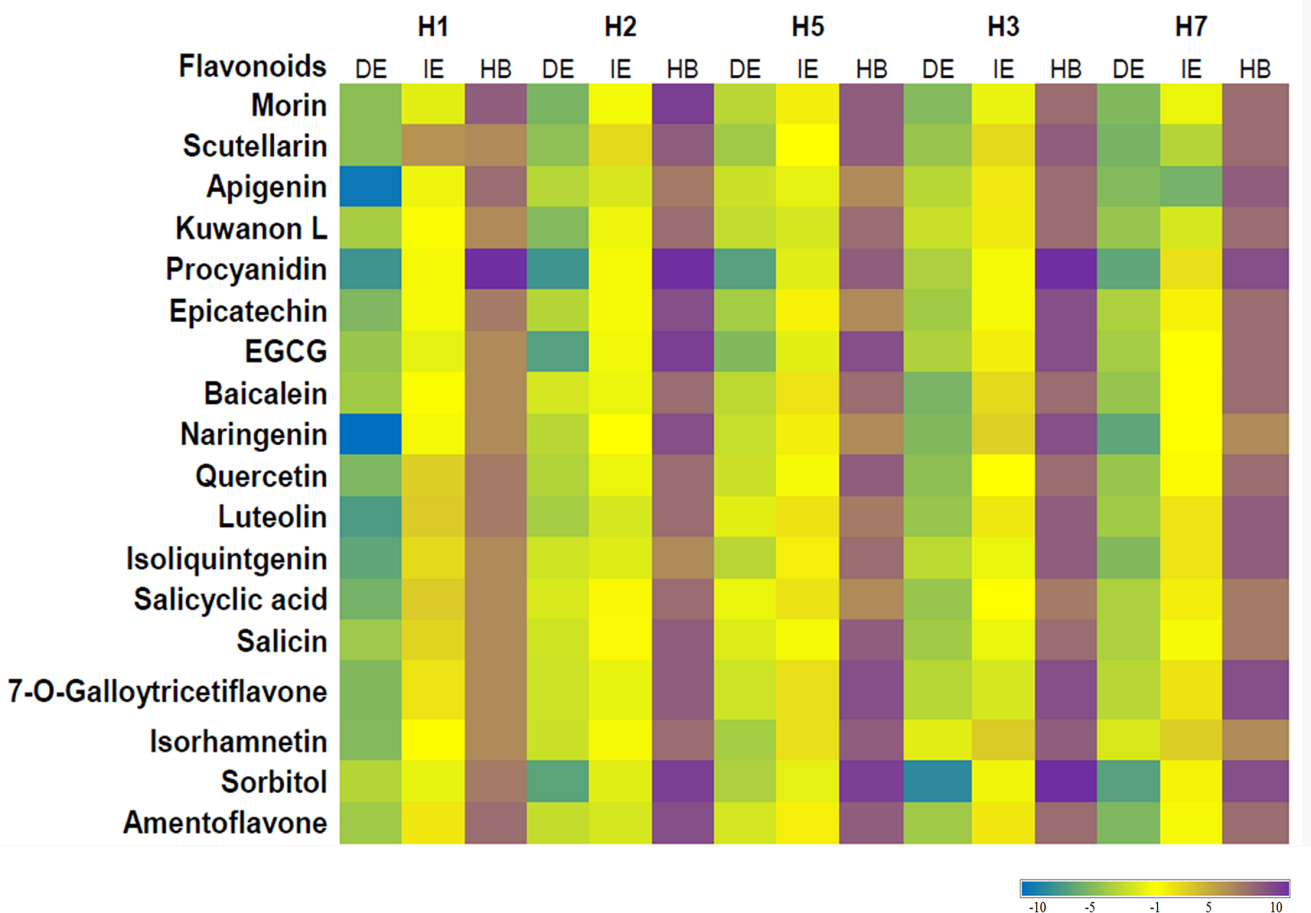
Heat map analysis of best 18 flavonoids binding to five HA subtypes while considering three main criteria: DE (Kcal/mol), HB and IE (Kcal/mol).

Almost all the selected ligands that bonded with H3 also reacted to H7 in the docking analysis. The top five predicted binders to H7 stem included sorbitol, procyanidin, 7-O-galloytricetiflavone, epicatechin and scutellarin (Fig 7). Sorbitol also showed robust binding abilities to H7 stem similar to H3. It bound with bNAb contact residues LYS38, GLY20, TRP21, THR41, LEU52, ILE56, and ALA292 with IE of -3.86 kcal·mol^−1^ and DE of -24.45 kcal·mol^−1^ (Fig 6B). Procyanidin and 7-O-galloytricetiflavone extended seven HB with an average of IE -2.66 kcal·mol^−1^ (Fig 7B and 7C). Morin, Kuwanon L, baicalein, quercetin, and amentoflavone principally formed five HB interactions with both H3 and H7 subtypes (S6 Table). Only naringenin and isorhamnetin formed three HB interaction with H7 stem while the rest 16 bioflavonoids formed almost five HB with the H7 stem. Least IE was observed with apigenin (IE:-17.61 kcal·mol^−1^, DE:-16.23 kcal·mol^−1^) and scutellarin (IE:-11.66 kcal·mol^−1^, DE:-17.39 kcal·mol^−1^) compared to all other flavonoids (S6 Table). Other flavonoids including luteolin, isoliquiritigenin, 7-O-galloytricetiflavone and isorhamnetin exhibited high IE, average of -2.84 kcal·mol^−1^ (S6 Table). Most of the screened 18 flavonoids exhibited binding at positions THR41, ASP46, THR49 and ILE56 of CR9114 bNAb epitope. These results indicated that sorbitol, procyanidin, and 7-O-galloytricetiflavone could intensively bind H3 and H7 stem epitope (Figs 5D, 5E, 7A, 7B and 7C). Heat map analysis of a number of HB and IE of best 18 bioflavonoids against group 1 and group 2 IAV are shown in Fig 8.

### Virtual screening study from bioassay hit compounds

**Fig 9:**
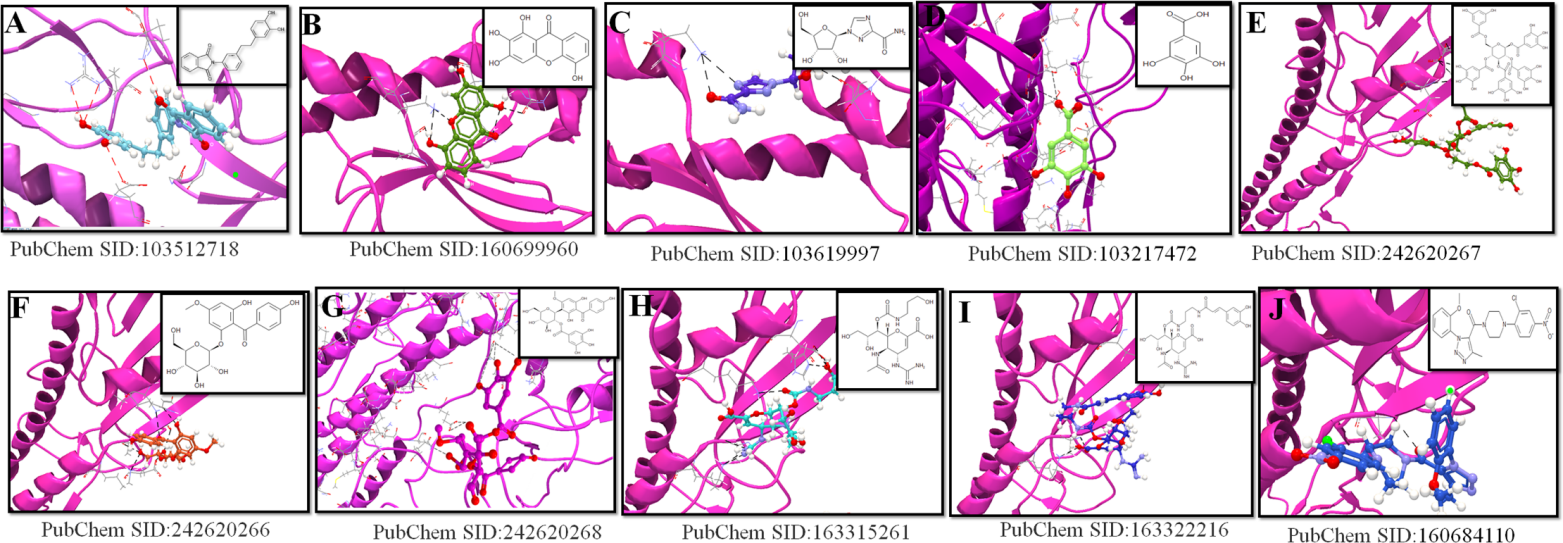
Virtual screening and molecular interaction with ten bioassay hit compounds on H1 stem region using CLC Drug Discovery Workbench. **This illustration shows interaction of ligands with maximum number of HB with the active site on the stem epitopes. H1 stem region is depicted in ribbon with HA1 and HA2 in pink color. HB interaction are represented in dashed black color.** (A) SID:103512718 (blue color), (B) SID:160699960 (green color), (C) SID:103619997 (dark blue color), (D) SID:103217472 (light blue color), (E) SID:242620267 (dark pale green color), (F) SID:242620266 (red color), (G) SID:242620268 (magenta color), (H) SID:163315261 (pale blue color), (I) SID:163322216 (purple color), and (J) SID:160684110 (pale dark blue color).

We analyzed 1413 potential compounds from bioassay hit molecules for best binding capabilities to CR6261 bNAb epitope on H1 HA stem. Ten compounds from PubChem, SID:103512718, SID:160699960, SID:103619997, SID:103217472, SID:242620267, SID:242620266, SID:242620268, SID:163315261, SID:163322216 and SID:160684110 extended best docking confirmation (Fig 9). The above ten molecules depicted a high number of HB but low IE and DE compared to flavonoids. Interestingly, the DE with bioassay hit molecules was lower (average:-7.45 kcal·mol^−1^) compared to the best DE from bioflavonoids (average:-16.57 kcal·mol^−1^) (S7 Table). Maximum number of four HB were formed with HA2 contact residues as recognized by alpha-helix of the CR6261 heavy chain. The contact residues of HA1 epitopes alpha-helix (LEU42, LEU292, VAL40, and HA2 epitopes ILE56, VAL52, and THR49) exhibited most of the binding (Fig 9). Interaction of binding with bioassay hit compounds were observed between adjacent HA2 monomers and the N-terminus of the fusion peptide, embedded in the hydrophobic pocket of the H1-HA stem. No interactions were observed at contact residues conserved in the hydrophobic tip with HIS8 and HIS28 from HA1 and ASN31 and LEU32 from HA2 (S7 Table). Virtual screened molecular interaction values of ten bioassays hit compounds docked with H1 stem region are depicted in Fig 9 with an average IE (-2.45 kcal·mol^−1^) and DE (-7.45 kcal·mol^−1^) (S7 Table).

## Discussion

Seasonal vaccines elicit neutralizing antibodies that recognize epitopes on the surface of the highly variable head region of HA. Accordingly, the vaccine components are varied every year in the hope of matching the circulating viral strains in the forthcoming influenza season. In the past several years, many bNAb that recognize the highly conserved stem region that encompasses the fusion peptide have been characterized [30]. The discovery of these antibodies opened new doors toward the development of what so-called “Universal Influenza Vaccine” that would protect from various subtypes and for a prolonged period of time [48].

On the other hand, with the continuous emergence of influenza strains that are resistant to the currently available anti-viral drugs such as Amantadine (anti-M2 proton) and Oseltamivir (Anti-NA), it becomes necessary to find alternative approaches to treat influenza. Here we report on a molecular docking study to screen for natural compounds binding potentials to the conserved influenza HA-stem that harbor the fusion peptide and is the prime target for bNAb. Two separate structure-based computational analysis were done. The first involved sequential docking analysis of 100 flavonoids for binding capabilities to the bNAb epitopes on both group 1 and group 2 influenza HA, and the other involved screening of 1413 compounds from bioassay hit molecules to identify their binding potentials to the H1-stem bNAb epitope. Our analysis improves upon others in two main aspects: firstly, we focused on the conserved epitopes which are the target of bNAb and secondly, we ran a sequential analysis by targeting HA-stem from various influenza subtypes. The analysis also included QSAR evaluation of the tested flavonoids to assess for their biological activities and physiochemical properties. ‎This enabled us to define a set of 18 broadly reactive compounds that exhibited significant HB interactions, high DE, and minimum IE with functional contact residues.

Procyanidin were the best binders to group1 HA stem with an average of eight HB, IE of -6.22 Kcal/mol and DE of -17.36 Kcal/mol. Second-inline potential binders for group1 HA stem included sorbitol (HB:7, IE:-5.63 Kcal/mol, DE:-15.63 Kcal/mol), morin (HB:6, IE:-4.68 Kcal/mol, DE:-14.12 Kcal/mol), EGCG (HB:7, IE:—4.63 Kcal/mol, DE:-15.11 Kcal/mol), quercetin (HB:5, IE:-3.63 Kcal/mol, DE:-14.63 Kcal/mol), kuwanon L (HB:6, IE:-3.09 Kcal/mol, DE:-13.15 Kcal/mol) and salicin (HB:5, IE:-4.20 Kcal/mol, DE:-12.79 Kcal/mol). In alignment with our findings, procyanidin have been recognized for their potent antiviral activity and immunostimulatory effects against IAV (Neutralization IC50 of 16.2–56.5 μg/ml) [49]. Derken *et*. *al* (2014) reported through *in silico* docking study the interaction of procyanidin with HA receptor binding site, thus, indicating its potential role in inhibiting virus attachment to host cells [50]. Further, procyanidin inhibited the growth of H1N1 clinical isolate was well as H1N1 mouse adapted strain, PR8 (IC_50_ of 2.2 μg/mL and 2.5 μg/mL, respectively) *in vitro* [51]. The ability of this compound to bind both, the receptor binding domain as well as the conserved stem epitope as demonstrated in our study, highlight its importance to develop next-generation broad anti-influenza drugs. The dimeric procyanidin has also shown significant inhibition against other viruses such as herpes simplex virus type 1 (HSV-1) [52].

Sorbitol was the best candidate for binding to group 2 IAV subtypes. Second-inline potential binders for group 2 HA stem included procyanidin (HB:8, IE:-4.23 Kcal/mol, DE:-17.43 Kcal/mol), EGCG (HB:6, IE:-3.76 Kcal/mol, DE:-11.56 Kcal/mol), epicatechin (HB:6, IE:-4.42 Kcal/mol, DE:-12.56Kcal/mol), 7-O-galloytricetiflavone (HB:7, IE:-4.65 Kcal/mol, DE:-11.53 Kcal/mol), and naringenin (HB:7, IE:-2.54 Kcal/mol, DE:-17.97 Kcal/mol). Although sorbitol was reported earlier for its antiviral properties, it was recommended for use as a cryoprotectant during lyophilization and freezing of several viruses such as a varicella-zoster virus, respiratory syncytial virus, HSV, cytomegalovirus, and 17D Yellow fever vaccine [53]. Other efficient binders for both group 1 and 2 IAV included EGCG, silybin, apigenin, naringenin, epicatechin, salicin, quercetin, scutellarin, baicalein 7-O-galloytricetiflavone and amentoflavone. The antiviral activity of EGCG against influenza virus was reported for the first time in 1993 [54]. The green tea molecule affected virus infectivity in cell culture and it was shown to agglutinate the influenza viruses, preventing the virus from absorbing to MDCK cells [54]. Quercetin, apigenin, naringenin, and silybin were recently evaluated for their anti-HSV-1 and anti-parainfluenza activities [55]. These compounds had high anti-HSV-1 activity with a minimum inhibitory concentration between 0.1 and 0.8 ug/mL with silybin and quercetin being the most effective [55]. Scutellarin, amentoflavone, and baicalein have been shown to inactivate different viruses at various levels depending on the virus type, concentration and the cell type used in the assay [56].

Many strains of influenza, including the 2009 H1N1 influenza, are now resistant to commercially available anti-influenza drugs such as Tamiflu (oseltamivir phosphate), Relenza (zanamivir), Flumadine (rimantadine) and others [57, 58]. Flavonoids appear to be an effective pharmacological expansion in the class of anti-viral complexes against IAV [59]. Although these natural flavonoids are poorly soluble and rapidly degraded by metabolism, they can be modified using structure-based design and nanotechnology-based approaches, to maintain high affinity binding while exhibiting long half-life and strong solubility. Incorporation of herbal drugs in the delivery system also gives aid to increase in solubility, enhance stability, protect from toxicity, enhance pharmacological activity, improve tissue macrophage distribution, improve the bioavailability, sustain delivery and enhance the targeting capacity of flavonoids [60]. Various nano-vehicles based drug delivery platforms including solid lipid nanoparticles [61], nanocarriers [62], nanocapsules [63], biodegradable nanoparticles [64], polymeric nanoparticles [65], polymeric micelles [66], dendrimers [67] and microemulsions [68] are emerging as viable alternatives that showed to selectively deliver flavonoids [57]. Certain dietary flavonoids such as quercetin [69], EGCG [70], and lycopene [71] have reached Phase I clinical trials for their pharmacological effects [72].

In conclusion, we identified 18 bioflavonoids capable to bind HA stem from different subtypes, thus could avert the HA conformational changes required to carry out its membranes attachment activity. Identified molecules from this study may be a new class of potent agents, but further confirmation in *in vitro* and *in vivo* is essential to design highly interactive universal IAV therapy.

## Supporting information

S1 Table The important contact residues within H1, H2, H5, H3 and H7 stem region as determined by complex crystal structure with bnAb. (DOCX)

S2 Table A total of hundred antiviral bioactive flavonoids were recruited for inhibition study against HA stem region of IAV group 1 subtypes of H1, H2, H5, and group 2 subtypes H3 and H7. (DOCX)

S3 Table QSAR predicted values and their applicability domain analysis for various models. (DOCX)

S4 Table Molecular docking study between bioflavonoids interacted with H1 HA stem region and their intermolecular docking values presented with interaction energy, Hbond energy, docking score, number of Hbond interaction and the interacting residues. (DOCX)

S5 Table Bioflavonoids and their docking score along with the number of Hbond formation in H2 and H5 HA stem region as obtained from molecular docking using AutoDock 4. (DOCX)

S6 Table Bioflavonoids and their docking score along with the number of Hbond formation in H3 and H7 HA stem region as obtained from molecular docking using AutoDock 4. (DOCX)

S7 Table Molecular interaction of ten bioassay hit compounds docked with H1 stem region. (DOCX)

S1 Fig HA structures of various HA subtypes with bnAb epitope on each structure shown in red. The HA trimers are colored in green with epitopes targeted by bnAb colored dark brown.
(TIF)

S2 Fig Crystal structure of CR6261 Fab in Complex with the 1918 H1N1 influenza virus hemagglutinin. Fab heavy and light chains and HA trimer are depicted in surface representation. Contact residues in the bNAb epitope are labeled in the right panel.
(TIF)

S3 Fig Crystal structure of C179 Fab in Complex with a H2N2 influenza virus hemagglutinin. Fab heavy and light chains and HA trimer are depicted in surface representation. Contact residues in the bNAb epitope are labeled in the right panel.
(TIF)

S4 Fig Crystal structure of F10 ScFv in complex with H5 influenza virus hemagglutinin. Fab heavy and light chains and HA trimer are depicted in surface representation. Contact residues in the bNAb epitope are labeled in the right panel.
(TIF)

S5 Fig Crystal structure of CR8020 Fab in complex with H3 influenza virus hemagglutinin. Fab heavy and light chains and HA trimer are depicted in surface representation. Contact residues in the bNAb epitope are labeled in the right panel.
(TIF)

S6 Fig Crystal structure of CR9114 Fab in complex with H7 influenza virus hemagglutinin. Fab heavy and light chains and HA trimer are depicted in surface representation. Contact residues in the bNAb epitope are labeled in the right panel.
(TIF)

S1 Video MP4 Video file. (MP4)
