# Supplementary material for: Computational screening of known broad-spectrum antiviral small organic molecules for potential influenza HA stem inhibitors
Source: PLoS One. 2018 Sep 4;13(9):e0203148. doi: 10.1371/journal.pone.0203148 (PMC6122827; doi:10.1371/journal.pone.0203148)
Supplement: S1 Table — (DOCX) [file pone.0203148.s001.docx]

| **Influenza Subtypes** | **bnAb Contact residues of stem epitope** | **References** |
| --- | --- | --- |
| H1 | HIS8, HIS28, VAL30, ASN31,LEU32, ASP70, GLY71, TRP72, THR92, GLN93, ILE96, ASN97, VAL103, ASN104, ILE107, THR100 | ([Ekiert et al., 2009](#_ENREF_21); [Yassine et al., 2015](#_ENREF_77)) |
| H2 | VAL18, ASP19, GLY20, LYS38, TRP21, THR41, PHE45, ASP46, LYS43, GLN42, HIS38, VAL52, ILE56, PRO293, THR291, LEU292, LYS40, THR318 | ([Dreyfus, Ekiert, & Wilson, 2013](#_ENREF_18)) |
| H5 | HIS38, GLY20, HIS18, ASP19, TYR102, GLN42, ILE45, MET54, PHE55, THR41 | ([Sui et al., 2009](#_ENREF_66)) |
| H3 | GLU15, GLY16, VAL18, ASP19, LEU318, GLU325, GLN34, ALA35, ALA36, ARG25, GLY33, THR32, ASN146, GLY150, GLU30 | ([Ekiert et al., 2011](#_ENREF_22)) |
| H7 | ILE18, ASP19, ALA36, LYS38, GLY20, TRP21, THR41, ILE45, GLN42, ASP46, THR49, SER40, LEU52, ILE56, ALA292, ARG291, VAL293, ILE45 | ([Dreyfus et al., 2012](#_ENREF_19)) |

**References:**

Dreyfus, C., Ekiert, D. C., & Wilson, I. A. (2013). Structure of a classical broadly neutralizing stem antibody in complex with a pandemic H2 influenza virus hemagglutinin. J Virol, 87(12), 7149-7154. doi:10.1128/jvi.02975-12

Dreyfus, C., Laursen, N. S., Kwaks, T., Zuijdgeest, D., Khayat, R., Ekiert, D. C., . . . Friesen, R. H. (2012). Highly conserved protective epitopes on influenza B viruses. Science, 337(6100), 1343-1348.

Ekiert, D. C., Bhabha, G., Elsliger, M. A., Friesen, R. H., Jongeneelen, M., Throsby, M., . . . Wilson, I. A. (2009). Antibody recognition of a highly conserved influenza virus epitope. Science, 324(5924), 246-251. doi:10.1126/science.1171491

Ekiert, D. C., Friesen, R. H., Bhabha, G., Kwaks, T., Jongeneelen, M., Yu, W., . . . Goudsmit, J. (2011). A highly conserved neutralizing epitope on group 2 influenza A viruses. Science, 333(6044), 843-850. doi:10.1126/science.1204839

Sui, J., Hwang, W. C., Perez, S., Wei, G., Aird, D., Chen, L. M., . . . Marasco, W. A. (2009). Structural and functional bases for broad-spectrum neutralization of avian and human influenza A viruses. Nat Struct Mol Biol, 16(3), 265-273. doi:10.1038/nsmb.1566

Yassine, H. M., Boyington, J. C., McTamney, P. M., Wei, C. J., Kanekiyo, M., Kong, W. P., . . . Graham, B. S. (2015). Hemagglutinin-stem nanoparticles generate heterosubtypic influenza protection. Nat Med, 21(9), 1065-1070. doi:10.1038/nm.3927
