# Supplementary material for: Computational screening of known broad-spectrum antiviral small organic molecules for potential influenza HA stem inhibitors
Source: PLoS One. 2018 Sep 4;13(9):e0203148. doi: 10.1371/journal.pone.0203148 (PMC6122827; doi:10.1371/journal.pone.0203148)
Supplement: S2 Table — (DOCX) [file pone.0203148.s002.docx]

| **References** | **Function in Viral step** | **Chemical Formula** | **Molecular weight (g/mol)** | **Bioflavonoids** | **S.No** |
| --- | --- | --- | --- | --- | --- |
| ([Moghadamtousi et al., 2014](#_ENREF_45)) | inhibitory activity against IMPDH process in VSV,FIPV,FHV,HSV,RSV | [C_21_H_20_O_6_](https://pubchem.ncbi.nlm.nih.gov/search/#collection=compounds&query_type=mf&query=C21H20O6&sort=mw&sort_dir=asc) | 368.385 | Cucurmin | 1 |
| ([Jiang et al., 2002](#_ENREF_31)) | inhibitory activities on the PI-3 virus in vitro by cytopathogenic effects (CPE) reduction assay | C22H28O6 | 388.46 | Caesalmin B | 2 |
| ([Wang, Yang, Yuan, Liu, & Liu, 2015](#_ENREF_66)) | Reduce the levels of steps subsequent to virus entry in RV, Prevent viral attachment, internalization and stimulate IFN secretion in HRSV | C20H18O7 | 370.357 | Glycyrrhiza Flavonol A | 3 |
| (Muhammed *Zayed et al.,*2016) | Antiviral activity | [C_19_H_15_NO_4_](https://pubchem.ncbi.nlm.nih.gov/search/#collection=compounds&query_type=mf&query=C19H15NO4&sort=mw&sort_dir=asc) | 321.332 | Griffithdione | 4 |
| ([Polyak, Ferenci, & Pawlotsky, 2013](#_ENREF_51)) | inhibit HCV infection in cell culture by variably blocking viral entry, viral fusion, viral RNA and protein synthesis, | C25H22O10 | 482.441 | Silybin B | 5 |
| ([S. G. Ma et al., 2013](#_ENREF_42)) | potent antiviral activity against coxsackie virus B3 and influenza virus A (H3N2) Unclear | C25H34O4 | 398.543 | Spirooliganone A | 6 |
| ([Steinmann, Buer, Pietschmann, & Steinmann, 2013](#_ENREF_61)) | a potent inhibitor human immunodeficiency virus, influenza A virus,HCV, Retroviridae, Orthomyxoviridae and Flaviviridae | [C_22_H_18_O_11_](https://pubchem.ncbi.nlm.nih.gov/search/#collection=compounds&query_type=mf&query=C22H18O11&sort=mw&sort_dir=asc) | 458.375 | (-)-Epigallocatechin gallate | 7 |
| ([Hafid et al., 2017](#_ENREF_28)) | inhibited viral entry process though a direct virucidal activity and targeting host cells | [C_17_H_14_O_6_](https://pubchem.ncbi.nlm.nih.gov/search/#collection=compounds&query_type=mf&query=C17H14O6&sort=mw&sort_dir=asc) | 314.293 | Ladanein | 8 |
| ([Tian, Li, Chai, Shah, & Gong, 2014](#_ENREF_62)) | activation of the antiviral RNA silencing pathway and the subsequent knockdown of the endogenous host gene in TRV | [C_40_H_56_O_3_](https://pubchem.ncbi.nlm.nih.gov/search/#collection=compounds&query_type=mf&query=C40H56O3&sort=mw&sort_dir=asc) | 584.885 | Capsanthin | 9 |
| ([Visintini Jaime et al., 2013](#_ENREF_65)) | Antiviral activity | [C_15_H_14_O_3_](https://pubchem.ncbi.nlm.nih.gov/search/#collection=compounds&query_type=mf&query=C15H14O3&sort=mw&sort_dir=asc) | 242.274 | Taraxacin | 10 |
| ([Naser, Bodinet, Tegtmeier, & Lindequist, 2005](#_ENREF_46)) | stimulatory and co-stimulatory effects on cytokine and antibody production and activation of macrophages and other immunocompetent cells, have been evaluated in numerous in vitro and in vivo | [C_10_H_16_O](https://pubchem.ncbi.nlm.nih.gov/search/#collection=compounds&query_type=mf&query=C10H16O&sort=mw&sort_dir=asc) | 152.237 | Thujone | 11 |
| ([Dong et al., 2014](#_ENREF_17)) | inhibited virus replication in  influenza A/WSN/33 infected A549 cells | C28H34O15 | 610.565 | Hesperidin | 12 |
| ([Venugopala, Rashmi, & Odhav, 2013](#_ENREF_64)) | **parainfluenza** type 3 virus [ | [C_10_H_8_O_4_](https://pubchem.ncbi.nlm.nih.gov/search/#collection=compounds&query_type=mf&query=C10H8O4&sort=mw&sort_dir=asc) | 192.17 | Scopoletin | 13 |
| ([Dayem, Choi, Kim, & Cho, 2015](#_ENREF_16)) | reduced virus-induced ROS generation and blocked cytoplasmic lysosome acidification and the lipidation of microtubule associated protein1 light chain 3-B in Influenza | C28H32O16 | 624.548 | Isorhamnetin | 14 |
| ([Dao et al., 2012](#_ENREF_15)) | IFA NA inhibitors | [C_25_H_28_O_15_](https://pubchem.ncbi.nlm.nih.gov/search/#collection=compounds&query_type=mf&query=C25H28O15&sort=mw&sort_dir=asc) | 568.484 | Polygalaxanthone III | 15 |
| ([Atta-ur-Rahman., 2003](#_ENREF_6)) | Antiviral activity | [C_25_H_26_O_6_](https://pubchem.ncbi.nlm.nih.gov/search/#collection=compounds&query_type=mf&query=C25H26O6&sort=mw&sort_dir=asc) | 422.477 | Sangenon | 16 |
| ([Chung, Chien, Huang, Yao, & Lee, 2014](#_ENREF_13)) | inhibited virus replication in H1N1 Tamiflu | [C_15_H_10_O_5_](https://pubchem.ncbi.nlm.nih.gov/search/#collection=compounds&query_type=mf&query=C15H10O5&sort=mw&sort_dir=asc) | 270.24 | Baicaein | 17 |
| ([Kim, Jeon, & Ko](#_ENREF_34)) | CHIKV in vitro replication inhibited | C15H12O5 | 272.256 | Naringenin | 18 |
| ([Ji et al., 2015](#_ENREF_30)) | Apigenin Restricts FMDV Infection and Inhibits Viral IRES | [C_15_H_10_O_5_](https://pubchem.ncbi.nlm.nih.gov/search/#collection=compounds&query_type=mf&query=C15H10O5&sort=mw&sort_dir=asc) | 270.24 | Apigenin | 19 |
| ([Johari, Kianmehr, Mustafa, Abubakar, & Zandi, 2012](#_ENREF_32)) | anti-dengue activity | [C_15_H_10_O_7_](https://pubchem.ncbi.nlm.nih.gov/search/#collection=compounds&query_type=mf&query=C15H10O7&sort=mw&sort_dir=asc) | 302.238 | Quercetin | 20 |
| ([Fan, Qian, Qian, & Li, 2016](#_ENREF_20)) | Antiviral activity of luteolin against Japanese encephalitis virus. | C15H10O6 | 286.239 | Luteolin | 21 |
| ([Amblard, Delinsky, Arbiser, & Schinazi, 2006](#_ENREF_3)) | antiviral and cytotoxic properties | C18H18O2 | 266.34 | Honokiol | 22 |
| ([S. C. Lin, Chen, Li, Lin, & Wang, 2017](#_ENREF_38)) | Antiviral activity of nobiletin against chikungunya virus in vitro | [C_21_H_22_O_8_](https://pubchem.ncbi.nlm.nih.gov/search/#collection=compounds&query_type=mf&query=C21H22O8&sort=mw&sort_dir=asc) | 402.399 | Nobelitin | 23 |
| ([L.-T. Lin et al., 2013](#_ENREF_36)) | Inhibits glycosaminoglycans for entry | [C_48_H_28_O_30_](https://pubchem.ncbi.nlm.nih.gov/search/#collection=compounds&query_type=mf&query=C48H28O30&sort=mw&sort_dir=asc) | 1084.722 | Punicalagin | 24 |
| ([Traboulsi et al., 2015](#_ENREF_63)) | Antiviral activity of isoliquiritigenin against influenza virus PR8/H1N1 in human bronchial epithelial cells. | [C_15_H_12_O_4_](https://pubchem.ncbi.nlm.nih.gov/search/#collection=compounds&query_type=mf&query=C15H12O4&sort=mw&sort_dir=asc) | 256.257 | Isoliquiritgenin | 25 |
| ([Gravina et al., 2011](#_ENREF_27)) | Antiviral effect of Haolanganmao Granules on influenza A virus | [C_22_H_22_O_11_](https://pubchem.ncbi.nlm.nih.gov/search/#collection=compounds&query_type=mf&query=C22H22O11&sort=mw&sort_dir=asc) | 462.407 | Homoplantagnin | 26 |
| ([Ghamali et al., 2016](#_ENREF_26)) | Antiviral activity | [C_22_H_22_O_12_](https://pubchem.ncbi.nlm.nih.gov/search/#collection=compounds&query_type=mf&query=C22H22O12&sort=mw&sort_dir=asc) | 478.406 | Nepitrin | 27 |
| ([Spivack, Prusoff, & Tritton, 1982](#_ENREF_60)) | Antiviral activity | [C_21_H_20_O_12_](https://pubchem.ncbi.nlm.nih.gov/search/#collection=compounds&query_type=mf&query=C21H20O12&sort=mw&sort_dir=asc) | 464.379 | 6-hydroxyluteolin 7-O-glycoside | 28 |
| ([Gravina et al., 2011](#_ENREF_27)) | Antiviral activity | [C_22_H_22_O_10_](https://pubchem.ncbi.nlm.nih.gov/search/#collection=compounds&query_type=mf&query=C22H22O10&sort=mw&sort_dir=asc) | 446.408 | naringenin-7-O-β-d-glucopyranoside | 29 |
| ([Song, Lee, & Seong, 2005](#_ENREF_59)) | Anti-infective & antiviral properties. | C15H14O6 | 290.271 | Catechin | 30 |
| ([Wu, Yang, Huang, Liu, & Wu, 2007](#_ENREF_69)) | antiviral activity of hyperoside | [C_21_H_20_O_12_](https://pubchem.ncbi.nlm.nih.gov/search/#collection=compounds&query_type=mf&query=C21H20O12&sort=mw&sort_dir=asc) | 464.379 | Hyperoside | 31 |
| ([Mitrocotsa, Mitaku, Axarlis, Harvala, & Malamas](#_ENREF_44)) | antiviral activity of kaempferol and its glycosides against human cytomegalovirus | C15H10O6 | 286.239 | kaempferol | 32 |
| ([Han et al., 2015](#_ENREF_29)) | Inhibitory effects of each compound at the different stages of DENV-2 infection | [C_27_H_30_O_16_](https://pubchem.ncbi.nlm.nih.gov/search/#collection=compounds&query_type=mf&query=C27H30O16&sort=mw&sort_dir=asc) | 610.521 | Rutin | 33 |
| ([Dao et al., 2012](#_ENREF_15)) | Antiviral activity | C15H10O4 | 254.241 | 3,4'-dihydroxyflavone | 34 |
| ([Özçelik, Aslan, Orhan, & Karaoglu, 2005](#_ENREF_49)) | Antiviral activity | C18H32O2 | 280.452 | Linoleic acid | 35 |
| ([Ahmed et al., 1995](#_ENREF_1)) | Antiviral activity | [C_8_H_15_NO_4_](https://pubchem.ncbi.nlm.nih.gov/search/#collection=compounds&query_type=mf&query=C8H15NO4&sort=mw&sort_dir=asc) | 189.211 | Castanospermine | 36 |
| ([Baba & Shigeta, 1987](#_ENREF_7)) | Antiviral activity |  |  | Glycyrrhizin | 37 |
|  | Antiviral activity | [C_21_H_18_O_7_](https://pubchem.ncbi.nlm.nih.gov/search/#collection=compounds&query_type=mf&query=C21H18O7&sort=mw&sort_dir=asc) | 382.368 | 5, 7, 4'-Trihydroxy-8-methoxyflavone | 38 |
| ([Floc'h & Werner, 1976](#_ENREF_21)) | antiviral activity | [C_6_H_13_NO_5_](https://pubchem.ncbi.nlm.nih.gov/search/#collection=compounds&query_type=mf&query=C6H13NO5&sort=mw&sort_dir=asc) | 179.172 | D-glucosamine | 39 |
| ([Spivack et al., 1982](#_ENREF_60)) | antiviral activity was masked by the cytotoxic effect on the host | C6H12O5 | 164.157 | 2-deoxy-D-glucose | 40 |
| ([Shokri, Katiraee, Fatahinia, & Minooeianhaghighi, 2017](#_ENREF_55)) | potently inhibit enterovirus 71 (EV71) by suppressing viral 3C protease (3Cpro) activity | [C_15_H_10_O_4_](https://pubchem.ncbi.nlm.nih.gov/search/#collection=compounds&query_type=mf&query=C15H10O4&sort=mw&sort_dir=asc) | 254.241 | Chrysin | 41 |
| ([Meyer, Afolayan, Taylor, & Erasmus, 1997](#_ENREF_43)) | Antiviral activity of galangin isolated from the aerial parts of Helichrysum aureonitens | C15H10O5 | 270.24 | Galangin | 42 |
| ([Mitrocotsa et al.](#_ENREF_44)) | shown to inhibit or decrease the activity of enzymes that partake in viral infection such as reverse transcriptase, viral proteases and neuraminidase | C15H10O6 | 286.239 | Kempferol | 43 |
| ([Gravina et al., 2011](#_ENREF_27)) | evaluated in vitro against equid herpesvirus 1 (EHV-1) by determining the virucidal activity and using the time of addition assay to test inhibition of the viral replication cycle | [C_15_H_10_O_7_](https://pubchem.ncbi.nlm.nih.gov/search/#collection=compounds&query_type=mf&query=C15H10O7&sort=mw&sort_dir=asc) | 302.238 | Morin | 44 |
| ([Sokmen et al., 2005](#_ENREF_58)) | exhibits inhibitory **activity** against a wide range of viruses | C16H12O6 | 300.266 | Dinatin | 45 |
| ([Zeng et al., 2013](#_ENREF_72)) | antiviral activity against respiratory syncytial virus ... | [C_21_H_20_O_10_](https://pubchem.ncbi.nlm.nih.gov/search/#collection=compounds&query_type=mf&query=C21H20O10&sort=mw&sort_dir=asc) | 432.381 | Vitexin | 46 |
| ([Cheng et al., 2013](#_ENREF_12)) | tested for their antiviral activity against porcine reproductive and respiratory syndrome virus (PRRSV) in vitro | [C_21_H_18_O_12_](https://pubchem.ncbi.nlm.nih.gov/search/#collection=compounds&query_type=mf&query=C21H18O12&sort=mw&sort_dir=asc) | 462.363 | Scutellarin | 47 |
| ([Fan et al., 2016](#_ENREF_20)) | Antiviral activity of luteolin against Japanese encephalitis virus. | [C_21_H_20_O_11_](https://pubchem.ncbi.nlm.nih.gov/search/#collection=compounds&query_type=mf&query=C21H20O11&sort=mw&sort_dir=asc) | 448.38 | Galuteolin | 48 |
| ([Johari et al., 2012](#_ENREF_32)) | Antiviral activity of baicalein and quercetin against the Japanese encephalitis virus | [C_16_H_12_O_6_](https://pubchem.ncbi.nlm.nih.gov/search/#collection=compounds&query_type=mf&query=C16H12O6&sort=mw&sort_dir=asc) | 300.266 | Rhamnocitrin | 49 |
| ([Kao, Wu, & Yen, 2014](#_ENREF_33)) | antiviral and antioxidant properties | [C_21_H_22_O_9_](https://pubchem.ncbi.nlm.nih.gov/search/#collection=compounds&query_type=mf&query=C21H22O9&sort=mw&sort_dir=asc) | 418.398 | Liquiiritin | 50 |
| ([Zhong et al., 2017](#_ENREF_73)) | antibacterial activities against Xoo and Rs and their antiviral activity against TMV were evaluated | [C_15_H_10_O_8_](https://pubchem.ncbi.nlm.nih.gov/search/#collection=compounds&query_type=mf&query=C15H10O8&sort=mw&sort_dir=asc) | 318.237 | Myricetin | 51 |
| ([Andres, Donovan, & Kuhlenschmidt, 2009](#_ENREF_5)) | Isoflavones and their related flavonoid compounds exert antiviral properties in vitro and in vivo against a wide range of viruses | [C_15_H_10_O_5_](https://pubchem.ncbi.nlm.nih.gov/search/#collection=compounds&query_type=mf&query=C15H10O5&sort=mw&sort_dir=asc) | 270.24 | Genistein | 52 |
| [Du *et al.*, 2003](#_ENREF_18)) | Antiviral activity against varicella-zoster virus (VZV) Antiviral activity against HIV (inhibition of cytopathic activity) Antiviral activity against encephalitis virus ... | [C_16_H_12_O_4_](https://pubchem.ncbi.nlm.nih.gov/search/#collection=compounds&query_type=mf&query=C16H12O4&sort=mw&sort_dir=asc) | 268.268 | Formononetin | 53 |
| ([Wei et al., 2015](#_ENREF_67)) | antiviral activity of ribavirin against influenza virus significantly | [C_15_H_10_O_4_](https://pubchem.ncbi.nlm.nih.gov/search/#collection=compounds&query_type=mf&query=C15H10O4&sort=mw&sort_dir=asc) | 254.241 | Daidzein | 54 |
| [Du *et al.*, 2003](#_ENREF_18)) | Inhibitory effects of each compound at the different stages of DENV-2 infection were examined using foci forming unit reduction assay (FFURA) and quantitative real-time polymerase chain amplification ... | [C_15_H_10_O_6_](https://pubchem.ncbi.nlm.nih.gov/search/#collection=compounds&query_type=mf&query=C15H10O6&sort=mw&sort_dir=asc) | 286.239 | Fisetin | 55 |
| ([Pleschka, Stein, Schoop, & Hudson, 2009](#_ENREF_50)) | Anti-viral properties and mode of action of standardized Echinacea purpurea extract against highly pathogenic avian influenza virus (H5N1, H7N7) and swine-origin H1N1 (S-OIV) | [C_15_H_10_O_5_](https://pubchem.ncbi.nlm.nih.gov/search/#collection=compounds&query_type=mf&query=C15H10O5&sort=mw&sort_dir=asc) | 270.24 | Sulphuretin | 56 |
| ([Shahat et al.](#_ENREF_54)) | significant inhibitory activity against herpes simplex virus type 1 (HSV-1) |  |  | Flavone glycoside | 57 |
| ([Du et al., 2003](#_ENREF_18)) | antiviral effects against herpes simplex virus type 1 and 2 (HSV-1 and HSV-2) at different potencies | [C_35_H_30_O_11_](https://pubchem.ncbi.nlm.nih.gov/search/#collection=compounds&query_type=mf&query=C35H30O11&sort=mw&sort_dir=asc) | 626.614 | Kuwanon L | 58 |
| ([Yao et al., 2016](#_ENREF_71)) | anti-inflammatory, antiviral and antimalarial activities, researchers have devoted much interest to its potential pharmaceutical value | [C_19_H_18_O_5_](https://pubchem.ncbi.nlm.nih.gov/search/#collection=compounds&query_type=mf&query=C19H18O5&sort=mw&sort_dir=asc) | 326.348 | Moracin | 49 |
| ([Yang et al., 2014](#_ENREF_70)) | antiviral activity against both influenza A and B viruses than the catechin monomers | [C_29_H_24_O_12_](https://pubchem.ncbi.nlm.nih.gov/search/#collection=compounds&query_type=mf&query=C29H24O12&sort=mw&sort_dir=asc) | 564.499 | Theaflavin | 60 |
| ([Yang et al., 2014](#_ENREF_70)) | antiviral activity against both influenza A and B viruses than the catechin monomers | C30H26O13 | 594.525 | Procyanidin | 61 |
| ([Schnitzler et al., 2010](#_ENREF_52)) | Antiviral activity and mode of action of propolis extracts and selected compounds | [C_15_H_12_O_2_](https://pubchem.ncbi.nlm.nih.gov/search/#collection=compounds&query_type=mf&query=C15H12O2&sort=mw&sort_dir=asc) | 224.259 | Flavanone | 62 |
| ([Nikolaeva-Glomb et al., 2014](#_ENREF_47)) | antiviral activity of total methanol extracts, as well as that of the anthocyanins and the non-anthocyanins from the following wild berries ... | C_15_H_11_O_2_^+^ | 223.251 | Anthrocyanidin | 63 |
| ([Amoros, Fauconnier, & Girre, 1987](#_ENREF_4)) | The antiviral activity of a triterpene saponin isolated from Anagallis arvensis, Primulaceae, was studied in vitro against several viruses including herpes simplex type 1, adenovirus type 6, vaccinia, vesicular stomatitis and poliovirus | C58H94O27 | 1223.363 | Saponin | 64 |
| ([Kim et al.](#_ENREF_34)) | Antiviral activity |  |  | hesperetin 7-O-(2″,6″-di-O-α-rhamnopyranosyl)-β-glucopyranoside | 65 |
| ([Pleschka et al., 2009](#_ENREF_50)) | Anti-viral properties and mode of action of standardized Echinacea purpurea extract against highly pathogenic avian influenza virus (H5N1, H7N7) and swine-origin H1N1 (S-OIV). | C35H42O20 | 782.701 | Matteflavoside | 66 |
| ([Chang, Wang, Yeh, Shieh, & Chiang, 2013](#_ENREF_11)) | anti-viral activity against human respiratory syncytial virus in human respiratory tract cell lines. | [C_20_H_24_N_2_OS](https://pubchem.ncbi.nlm.nih.gov/search/#collection=compounds&query_type=mf&query=C20H24N2OS&sort=mw&sort_dir=asc) | 340.485 | Cinaserin | 67 |
| ([Song et al., 2005](#_ENREF_59)) | Antiviral effect of catechins in green tea on influenza virus. | [C_15_H_14_O_6_](https://pubchem.ncbi.nlm.nih.gov/search/#collection=compounds&query_type=mf&query=C15H14O6&sort=mw&sort_dir=asc) | 290.271 | Epicatechins | 68 |
| ([Serkedjieva & Velcheva, 2003](#_ENREF_53)) | unclear | [C_21_H_23_NO_5_](https://pubchem.ncbi.nlm.nih.gov/search/#collection=compounds&query_type=mf&query=C21H23NO5&sort=mw&sort_dir=asc) | 369.417 | Thalimonine | 69 |
| ([Liu, Hu, Shen, Wang, & Zhu, 2017](#_ENREF_40)) | antiviral activity of coumarin derivatives against spring viraemia of carp virus in epithelioma papulosum cyprini cells. | C9H6O2 | 146.145 | Coumarins | 70 |
| ([Nothias-Scaglia et al., 2015](#_ENREF_48)) | Antiviral Activity of Diterpene Esters on Chikungunya Virus and HIV Replication | [C_20_H_36_O_3_](https://pubchem.ncbi.nlm.nih.gov/search/#collection=compounds&query_type=mf&query=C20H36O3&sort=mw&sort_dir=asc) | 324.505 | Diterpene | 71 |
| ([Fukuchi et al., 2016](#_ENREF_23)) | anti-viral and antitumor substances from natural resources, antiviral and antitumor activities of licorice root extract and purified ingredients were investigated. | C42H62O16Zn | 888.322 | Licorice | 72 |
| ([Shahat et al.](#_ENREF_54)) | The antiviral and antioxidant activity of some fractions and of a series of flavonoids and proanthocyanidins obtained from Crataegus sinaica (Rosaceae) | C29H38O4 | 450.619 | Triterpene | 73 |
| ([Evers et al., 2005](#_ENREF_19)) | antiviral activity against human cytomegalovirus for certain dietary flavonoids and their likely biochemical mechanisms of action. | [C_16_H_12_O_5_](https://pubchem.ncbi.nlm.nih.gov/search/#collection=compounds&query_type=mf&query=C16H12O5&sort=mw&sort_dir=asc) | 284.267 | Biochanin | 74 |
| ([Johari et al., 2012](#_ENREF_32)) | Antiviral activity of baicalein and quercetin against the Japanese encephalitis virus. | C15H10O5 | 270.24 | Baicalein | 75 |
| ([Pleschka et al., 2009](#_ENREF_50)) | Anti-viral properties and mode of action of standardized Echinacea purpurea extract against highly pathogenic avian influenza virus (H5N1, H7N7) and swine-origin H1N1 (S-OIV). | [C_16_H_12_O_6_](https://pubchem.ncbi.nlm.nih.gov/search/#collection=compounds&query_type=mf&query=C16H12O6&sort=mw&sort_dir=asc) | 300.266 | Isocutellarein | 76 |
| ([L. T. Lin, Hsu, & Lin, 2014](#_ENREF_37)) | antiviral activity against HCoV-22E9 | [C_18_H_22_N_2_O_4_](https://pubchem.ncbi.nlm.nih.gov/search/#collection=compounds&query_type=mf&query=C18H22N2O4&sort=mw&sort_dir=asc) | 330.384 | Quinocarcin | 77 |
| ([Pleschka et al., 2009](#_ENREF_50)) | Antiviral activity | C14H12O8 | 308.242 | Fulvic acid | 78 |
| ([S. C. Ma et al., 2001](#_ENREF_41)) | antiviral activity against respiratory syncytial virus (RSV), with an IC50 of 5.5 microg/ml. | C30H18O10 | 538.464 | Amentoflavone | 79 |
| ([Freitas et al., 2009](#_ENREF_22)) | Antiviral activity-guided fractionation from Araucaria angustifolia leaves extract | C30H18O10 | 538.464 | Robustaflavone | 80 |
| ([Pleschka et al., 2009](#_ENREF_50)) | Antiviral activity | C30H18O10 | 538.464 | Agatisflavone | 81 |
| ([Pleschka et al., 2009](#_ENREF_50)) | Antiviral activity | C20H20O6 | 356.374 | Laurifolin | 82 |
| ([Lisov, Vrublevskaya, Lisova, Leontievsky, & Morenkov, 2015](#_ENREF_39)) | antiviral activity against PRV (IC50, 1.5–15 µg/mL for different virus strains) and BoHV-1 (IC50, 0.5–0.7 µg/mL). |  |  | Elatin | 83 |
| ([Likhitwitayawuid et al., 2005](#_ENREF_35)) | Antiviral activity | C21H20O4 | 336.387 | Lanceolatin | 84 |
| [Du *et al.*, 2003](#_ENREF_18)) | Antiviral activity | C30H48O4 | 472.71 | Hispidone | 85 |
| ([Berlutti et al., 2011](#_ENREF_8)) | Antiviral properties of lactoferrin--a natural immunity molecule | [C_18_H_16_O_8_](https://pubchem.ncbi.nlm.nih.gov/search/#collection=compounds&query_type=mf&query=C18H16O8&sort=mw&sort_dir=asc) | 360.318 | Eupatin | 86 |
| [Du *et al.*, 2003](#_ENREF_18)) | Antiviral activity | [C_20_H_20_O_4_](https://pubchem.ncbi.nlm.nih.gov/search/#collection=compounds&query_type=mf&query=C20H20O4&sort=mw&sort_dir=asc) | 324.376 | Glabranin | 87 |
| ([Meyer et al., 1997](#_ENREF_43)) | Antiviral activity of galangin isolated from the aerial parts of Helichrysum aureonitens. |  |  | Galangin | 88 |
| ([Simöes, Amoros, Girre, Gleye, & Fauvel, 1990](#_ENREF_56)) | ANTIVIRAL ACTIVITY OF TERNATIN AND MELITERNATIN,. 3-METHOXYFLAVONES FROM SPECIES OF RUTACEAE'. C. M. 0. SIMOES | [C_19_H_18_O_8_](https://pubchem.ncbi.nlm.nih.gov/search/#collection=compounds&query_type=mf&query=C19H18O8&sort=mw&sort_dir=asc) | 374.345 | Ternatin | 89 |
| ([Singh, Moore, Gilliland, & Carr, 2004](#_ENREF_57)) | Antiviral defense mechanisms by salicylic acid. | [C_7_H_6_O_3_](https://pubchem.ncbi.nlm.nih.gov/search/#collection=compounds&query_type=mf&query=C7H6O3&sort=mw&sort_dir=asc) | 138.122 | Salicyclic acid | 90 |
| ([Alche, Barquero, Sanjuan, & Coto, 2002](#_ENREF_2)) | antiviral activity against herpes simplex virus type 1 (HSV-1) by inhibiting specific infected-cell polypeptides (ICPs) produced late in infection | C21H18O8 | 398.367 | Efinrin | 91 |
| [Du *et al.*, 2003](#_ENREF_18)) | Antiviral activity | [C_20_H_28_O_3_](https://pubchem.ncbi.nlm.nih.gov/search/#collection=compounds&query_type=mf&query=C20H28O3&sort=mw&sort_dir=asc) | 316.441 | Gibbercellin | 92 |
| ([Welch et al., 1985](#_ENREF_68)) | antiviral properties of an acyclovir-phospholipid conjugate |  |  | Phosphatidic acid | 93 |
| ([Dall’Acqua et al., 2011](#_ENREF_14)) | Antiviral activity | [C_22_H_30_O_4_](https://pubchem.ncbi.nlm.nih.gov/search/#collection=compounds&query_type=mf&query=C22H30O4&sort=mw&sort_dir=asc) | 358.478 | Ferutinin | 94 |
| ([Calland, Dubuisson, Rouille, & Seron, 2012](#_ENREF_10)) | Antiviral activity in primary human hepatocytes, but with an increased IC50 (10 µM). | C13H18O7 | 286.28 | Salicin | 95 |
| ([Gao et al., 2015](#_ENREF_24)) | Antiviral activity | C6H14O6 | 182.172 | Sorbitol | 96 |
| [Du *et al.*, 2003](#_ENREF_18)) | Antiviral activity | [C_17_H_13_NO_3_](https://pubchem.ncbi.nlm.nih.gov/search/#collection=compounds&query_type=mf&query=C17H13NO3&sort=mw&sort_dir=asc) | 279.295 | Graveoline | 97 |
| ([Brinkevich, Boreko, Savinova, Pavlova, & Shadyro, 2012](#_ENREF_9)) | Radical-regulating and antiviral properties of ascorbic acid and its derivatives | [C_6_H_8_O_6_](https://pubchem.ncbi.nlm.nih.gov/search/#collection=compounds&query_type=mf&query=C6H8O6&sort=mw&sort_dir=asc) | 176.124 | Ascorbic acid | 98 |
| [Du *et al.*, 2003](#_ENREF_18)) | Antiviral activity | [C_35_H_38_N_2_O_8_](https://pubchem.ncbi.nlm.nih.gov/search/#collection=compounds&query_type=mf&query=C35H38N2O8&sort=mw&sort_dir=asc) | 614.695 | Pendulinin | 99 |
| ([Gaudineau & Auclair, 2004](#_ENREF_25)) | Nicotinic acid has been used as a cholesterol-lowering agent for a few decades already, whereas the cytoprotective and antiviral properties of nicotinamide are slowly gaining attention | C6H5NO2 | 123.111 | Nicotinic acid | 100 |

**References:**

Ahmed, S. P., Nash, R. J., Bridges, C. G., Taylor, D. L., Kang, M. S., Porter, E. A., & Tyms, A. S. (1995). Antiviral activity and metabolism of the castanospermine derivative MDL 28,574, in cells infected with herpes simplex virus type 2. *Biochem Biophys Res Commun, 208*(1), 267-273.

Alche, L. E., Barquero, A. A., Sanjuan, N. A., & Coto, C. E. (2002). An antiviral principle present in a purified fraction from Melia azedarach L. leaf aqueous extract restrains herpes simplex virus type 1 propagation. *Phytother Res, 16*(4), 348-352.

Amblard, F., Delinsky, D., Arbiser, J. L., & Schinazi, R. F. (2006). Facile purification of honokiol and its antiviral and cytotoxic properties. *J Med Chem, 49*(11), 3426-3427.

Amoros, M., Fauconnier, B., & Girre, R. L. (1987). In vitro antiviral activity of a saponin from Anagallis arvensis, Primulaceae, against herpes simplex virus and poliovirus. *Antiviral Res, 8*(1), 13-25.

Andres, A., Donovan, S. M., & Kuhlenschmidt, M. S. (2009). Soy isoflavones and virus infections. *J Nutr Biochem, 20*(8), 563-569.

Atta-ur-Rahman. (2003). *Studies in Natural Products Chemistry Volume 29, Part J, Pages 3-902 (2003). Bioactive Natural Products (Part J). Edited by Atta-ur-Rahman.*

Baba, M., & Shigeta, S. (1987). Antiviral activity of glycyrrhizin against varicella-zoster virus in vitro. *Antiviral Res, 7*(2), 99-107.

Berlutti, F., Pantanella, F., Natalizi, T., Frioni, A., Paesano, R., Polimeni, A., & Valenti, P. (2011). Antiviral properties of lactoferrin--a natural immunity molecule. *Molecules, 16*(8), 6992-7018.

Brinkevich, S. D., Boreko, E. I., Savinova, O. V., Pavlova, N. I., & Shadyro, O. I. (2012). Radical-regulating and antiviral properties of ascorbic acid and its derivatives. *Bioorg Med Chem Lett, 22*(7), 2424-2427.

Calland, N., Dubuisson, J., Rouille, Y., & Seron, K. (2012). Hepatitis C virus and natural compounds: a new antiviral approach? *Viruses, 4*(10), 2197-2217.

Chang, J. S., Wang, K. C., Yeh, C. F., Shieh, D. E., & Chiang, L. C. (2013). Fresh ginger (Zingiber officinale) has anti-viral activity against human respiratory syncytial virus in human respiratory tract cell lines. *J Ethnopharmacol, 145*(1), 146-151.

Cheng, J., Sun, N., Zhao, X., Niu, L., Song, M., Sun, Y., . . . Li, H. (2013). In vitro screening for compounds derived from traditional chinese medicines with antiviral activities against porcine reproductive and respiratory syndrome virus. *J Microbiol Biotechnol, 23*(8), 1076-1083.

Chung, S. T., Chien, P. Y., Huang, W. H., Yao, C. W., & Lee, A. R. (2014). Synthesis and anti-influenza activities of novel baicalein analogs. *Chem Pharm Bull, 62*(5), 415-421.

Dall’Acqua, S., Linardi, M. A., Maggi, F., Nicoletti, M., Petitto, V., Innocenti, G., . . . Viola, G. (2011). Natural daucane sesquiterpenes with antiproliferative and proapoptotic activity against human tumor cells. *Bioorganic & Medicinal Chemistry, 19*(19), 5876-5885. doi:<https://doi.org/10.1016/j.bmc.2011.08.021>

Dao, T. T., Dang, T. T., Nguyen, P. H., Kim, E., Thuong, P. T., & Oh, W. K. (2012). Xanthones from Polygala karensium inhibit neuraminidases from influenza A viruses. *Bioorg Med Chem Lett, 22*(11), 3688-3692. doi:10.1016/j.bmcl.2012.04.028

Dayem, A. A., Choi, H. Y., Kim, Y. B., & Cho, S.-G. (2015). Antiviral Effect of Methylated Flavonol Isorhamnetin against Influenza. *PLOS ONE, 10*(3), e0121610. doi:10.1371/journal.pone.0121610

Dong, W., Wei, X., Zhang, F., Hao, J., Huang, F., Zhang, C., & Liang, W. (2014). A dual character of flavonoids in influenza A virus replication and spread through modulating cell-autonomous immunity by MAPK signaling pathways. *Scientific Reports, 4*, 7237. doi:10.1038/srep07237

Du, J., He, Z. D., Jiang, R. W., Ye, W. C., Xu, H. X., & But, P. P. (2003). Antiviral flavonoids from the root bark of Morus alba L. *Phytochemistry, 62*(8), 1235-1238.

Evers, D. L., Chao, C.-F., Wang, X., Zhang, Z., Huong, S.-M., & Huang, E.-S. (2005). Human cytomegalovirus-inhibitory flavonoids: Studies on antiviral activity and mechanism of action. *Antiviral Research, 68*(3), 124-134. doi:<https://doi.org/10.1016/j.antiviral.2005.08.002>

Fan, W., Qian, S., Qian, P., & Li, X. (2016). Antiviral activity of luteolin against Japanese encephalitis virus. *Virus Res, 220*, 112-116.

Floc'h, F., & Werner, G. H. (1976). In vivo antiviral activity of D-glucosamine. *Arch Virol, 52*(1-2), 169-173.

Freitas, A. M., Almeida, M. T., Andrighetti-Frohner, C. R., Cardozo, F. T., Barardi, C. R., Farias, M. R., & Simoes, C. M. (2009). Antiviral activity-guided fractionation from Araucaria angustifolia leaves extract. *J Ethnopharmacol, 126*(3), 512-517.

Fukuchi, K., Okudaira, N., Adachi, K., Odai-Ide, R., Watanabe, S., Ohno, H., . . . Sakagami, H. (2016). Antiviral and Antitumor Activity of Licorice Root Extracts. *In Vivo, 30*(6), 777-785.

Gao, M. J., Zhan, X. B., Gao, P., Zhang, X., Dong, S. J., Li, Z., . . . Lin, C. C. (2015). Improving Performance and Operational Stability of Porcine Interferon-alpha Production by Pichia pastoris with Combinational Induction Strategy of Low Temperature and Methanol/Sorbitol Co-feeding. *Appl Biochem Biotechnol, 176*(2), 493-504.

Gaudineau, C., & Auclair, K. (2004). Inhibition of human P450 enzymes by nicotinic acid and nicotinamide. *Biochem Biophys Res Commun, 317*(3), 950-956.

Ghamali, M., Chtita, S., Hmamouchi, R., Adad, A., Bouachrine, M., & Lakhlifi, T. (2016). The inhibitory activity of aldose reductase of flavonoid compounds: Combining DFT and QSAR calculations. *Journal of Taibah University for Science, 10*(4), 534-542. doi:<https://doi.org/10.1016/j.jtusci.2015.09.006>

Gravina, H. D., Tafuri, N. F., Silva Júnior, A., Fietto, J. L. R., Oliveira, T. T., Diaz, M. A. N., & Almeida, M. R. (2011). In vitro assessment of the antiviral potential of trans-cinnamic acid, quercetin and morin against equid herpesvirus 1. *Research in Veterinary Science, 91*(3), e158-e162. doi:<https://doi.org/10.1016/j.rvsc.2010.11.010>

Hafid, A. F., Aoki-Utsubo, C., Permanasari, A. A., Adianti, M., Tumewu, L., Widyawaruyanti, A., . . . Hotta, H. (2017). Antiviral activity of the dichloromethane extracts from Artocarpus heterophyllus leaves against hepatitis C virus. *Asian Pacific Journal of Tropical Biomedicine, 7*(7), 633-639. doi:<http://dx.doi.org/10.1016/j.apjtb.2017.06.003>

Han, Y., Ding, Y., Xie, D., Hu, D., Li, P., Li, X., . . . Song, B. (2015). Design, synthesis, and antiviral activity of novel rutin derivatives containing 1, 4-pentadien-3-one moiety. *Eur J Med Chem, 92*, 732-737.

Ji, P., Chen, C., Hu, Y., Zhan, Z., Pan, W., Li, R., . . . Yang, G. (2015). Antiviral activity of Paulownia tomentosa against enterovirus 71 of hand, foot, and mouth disease. *Biol Pharm Bull, 38*(1), 1-6.

Jiang, R. W., Ma, S. C., He, Z. D., Huang, X. S., But, P. P., Wang, H., . . . Mak, T. C. (2002). Molecular structures and antiviral activities of naturally occurring and modified cassane furanoditerpenoids and friedelane triterpenoids from Caesalpinia minax. *Bioorg Med Chem, 10*(7), 2161-2170.

Johari, J., Kianmehr, A., Mustafa, M. R., Abubakar, S., & Zandi, K. (2012). Antiviral activity of baicalein and quercetin against the Japanese encephalitis virus. *Int J Mol Sci, 13*(12), 16785-16795.

Kao, T.-C., Wu, C.-H., & Yen, G.-C. (2014). Bioactivity and Potential Health Benefits of Licorice. *Journal of Agricultural and Food Chemistry, 62*(3), 542-553. doi:10.1021/jf404939f

Kim, H. K., Jeon, W. K., & Ko, B. S. *Flavanone glycosides from Citrus junos and their anti-influenza virus activity*: Planta Med. 2001 Aug;67(6):548-9.

Likhitwitayawuid, K., Sritularak, B., Benchanak, K., Lipipun, V., Mathew, J., & Schinazi, R. F. (2005). Phenolics with antiviral activity from Millettia erythrocalyx and Artocarpus lakoocha. *Nat Prod Res, 19*(2), 177-182.

Lin, L.-T., Chen, T.-Y., Lin, S.-C., Chung, C.-Y., Lin, T.-C., Wang, G.-H., . . . Richardson, C. D. (2013). Broad-spectrum antiviral activity of chebulagic acid and punicalagin against viruses that use glycosaminoglycans for entry. *BMC Microbiology, 13*(1), 187. doi:10.1186/1471-2180-13-187

Lin, L. T., Hsu, W. C., & Lin, C. C. (2014). Antiviral natural products and herbal medicines. *J Tradit Complement Med, 4*(1), 24-35.

Lin, S. C., Chen, M. C., Li, S., Lin, C. C., & Wang, T. T. (2017). Antiviral activity of nobiletin against chikungunya virus in vitro. *Antivir Ther, 13*(10).

Lisov, A., Vrublevskaya, V., Lisova, Z., Leontievsky, A., & Morenkov, O. (2015). A 2,5-Dihydroxybenzoic Acid-Gelatin Conjugate: The Synthesis, Antiviral Activity and Mechanism of Antiviral Action Against Two Alphaherpesviruses. *Viruses, 7*(10), 5343-5360.

Liu, L., Hu, Y., Shen, Y. F., Wang, G. X., & Zhu, B. (2017). Evaluation on antiviral activity of coumarin derivatives against spring viraemia of carp virus in epithelioma papulosum cyprini cells. *Antiviral Res, 144*, 173-185.

Ma, S. C., But, P. P., Ooi, V. E., He, Y. H., Lee, S. H., Lee, S. F., & Lin, R. C. (2001). Antiviral amentoflavone from Selaginella sinensis. *Biol Pharm Bull, 24*(3), 311-312.

Ma, S. G., Gao, R. M., Li, Y. H., Jiang, J. D., Gong, N. B., Li, L., . . . Yu, S. S. (2013). Antiviral spirooliganones A and B with unprecedented skeletons from the roots of Illicium oligandrum. *Org Lett, 15*(17), 4450-4453.

Meyer, J. J., Afolayan, A. J., Taylor, M. B., & Erasmus, D. (1997). Antiviral activity of galangin isolated from the aerial parts of Helichrysum aureonitens. *J Ethnopharmacol, 56*(2), 165-169.

Mitrocotsa, D., Mitaku, S., Axarlis, S., Harvala, C., & Malamas, M. *Evaluation of the antiviral activity of kaempferol and its glycosides against human cytomegalovirus*: Planta Med. 2000 May;66(4):377-9. doi: 10.1055/s-2000-8550.

Moghadamtousi, S. Z., Kadir, H. A., Hassandarvish, P., Tajik, H., Abubakar, S., & Zandi, K. (2014). A review on antibacterial, antiviral, and antifungal activity of curcumin. *Biomed Res Int, 2014*, 186864. doi:10.1155/2014/186864

Naser, B., Bodinet, C., Tegtmeier, M., & Lindequist, U. (2005). Thuja occidentalis (Arbor vitae): A Review of its Pharmaceutical, Pharmacological and Clinical Properties. *Evidence-based Complementary and Alternative Medicine, 2*(1), 69-78. doi:10.1093/ecam/neh065

Nikolaeva-Glomb, L., Mukova, L., Nikolova, N., Badjakov, I., Dincheva, I., Kondakova, V., . . . Galabov, A. S. (2014). In vitro antiviral activity of a series of wild berry fruit extracts against representatives of Picorna-, Orthomyxo- and Paramyxoviridae. *Nat Prod Commun, 9*(1), 51-54.

Nothias-Scaglia, L. F., Pannecouque, C., Renucci, F., Delang, L., Neyts, J., Roussi, F., . . . Paolini, J. (2015). Antiviral Activity of Diterpene Esters on Chikungunya Virus and HIV Replication. *J Nat Prod, 78*(6), 1277-1283.

Özçelik, B., Aslan, M., Orhan, I., & Karaoglu, T. (2005). Antibacterial, antifungal, and antiviral activities of the lipophylic extracts of Pistacia vera. *Microbiological Research, 160*(2), 159-164. doi:<https://doi.org/10.1016/j.micres.2004.11.002>

Pleschka, S., Stein, M., Schoop, R., & Hudson, J. B. (2009). Anti-viral properties and mode of action of standardized Echinacea purpurea extract against highly pathogenic avian influenza virus (H5N1, H7N7) and swine-origin H1N1 (S-OIV). *Virol J, 6*(197), 6-197.

Polyak, S. J., Ferenci, P., & Pawlotsky, J.-M. (2013). Hepatoprotective and Antiviral Functions of Silymarin Components in HCV Infection. *Hepatology (Baltimore, Md.), 57*(3), 1262-1271. doi:10.1002/hep.26179

Schnitzler, P., Neuner, A., Nolkemper, S., Zundel, C., Nowack, H., Sensch, K. H., & Reichling, J. (2010). Antiviral activity and mode of action of propolis extracts and selected compounds. *Phytother Res, 24*(1).

Serkedjieva, J., & Velcheva, M. (2003). In vitro anti-influenza virus activity of the pavine alkaloid (-)-thalimonine isolated from Thalictrum simplex L. *Antivir Chem Chemother, 14*(2), 75-80.

Shahat, A. A., Cos, P., De Bruyne, T., Apers, S., Hammouda, F. M., Ismail, S. I., . . . Vlietinck, A. J. *Antiviral and antioxidant activity of flavonoids and proanthocyanidins from Crataegus sinaica*: Planta Med. 2002 Jun;68(6):539-41. doi: 10.1055/s-2002-32547.

Shokri, H., Katiraee, F., Fatahinia, M., & Minooeianhaghighi, M. H. (2017). Chemical composition and antifungal potential of Iranian propolis against Candida krusei strains. *Journal of Apicultural Research, 56*(5), 581-587. doi:10.1080/00218839.2017.1371534

Simöes, C. M. O., Amoros, M., Girre, L., Gleye, J., & Fauvel, M. T. (1990). Antiviral Activity of Ternatin and Meliternatin, 3-Methoxyflavones from Species of Rutaceae. *Journal of Natural Products, 53*(4), 989-992. doi:10.1021/np50070a036

Singh, D. P., Moore, C. A., Gilliland, A., & Carr, J. P. (2004). Activation of multiple antiviral defence mechanisms by salicylic acid. *Mol Plant Pathol, 5*(1), 57-63.

Sokmen, M., Angelova, M., Krumova, E., Pashova, S., Ivancheva, S., Sokmen, A., & Serkedjieva, J. (2005). In vitro antioxidant activity of polyphenol extracts with antiviral properties from Geranium sanguineum L. *Life Sciences, 76*(25), 2981-2993. doi:<https://doi.org/10.1016/j.lfs.2004.11.020>

Song, J. M., Lee, K. H., & Seong, B. L. (2005). Antiviral effect of catechins in green tea on influenza virus. *Antiviral Res, 68*(2), 66-74.

Spivack, J. G., Prusoff, W. H., & Tritton, T. R. (1982). A study of the antiviral mechanism of action of 2-deoxy-D-glucose: normally glycosylated proteins are not strictly required for herpes simplex virus attachment but increase viral penetration and infectivity. *Virology, 123*(1), 123-138.

Steinmann, J., Buer, J., Pietschmann, T., & Steinmann, E. (2013). Anti-infective properties of epigallocatechin-3-gallate (EGCG), a component of green tea. *British Journal of Pharmacology, 168*(5), 1059-1073. doi:10.1111/bph.12009

Tian, S.-L., Li, L., Chai, W.-G., Shah, S. N. M., & Gong, Z.-H. (2014). Effects of silencing key genes in the capsanthin biosynthetic pathway on fruit color of detached pepper fruits. *BMC Plant Biology, 14*(1), 314. doi:10.1186/s12870-014-0314-3

Traboulsi, H., Cloutier, A., Boyapelly, K., Bonin, M. A., Marsault, E., Cantin, A. M., & Richter, M. V. (2015). The Flavonoid Isoliquiritigenin Reduces Lung Inflammation and Mouse Morbidity during Influenza Virus Infection. *Antimicrob Agents Chemother, 59*(10), 6317-6327.

Venugopala, K. N., Rashmi, V., & Odhav, B. (2013). Review on natural coumarin lead compounds for their pharmacological activity. *Biomed Res Int, 963248*(10), 24.

Visintini Jaime, M. F., Redko, F., Muschietti, L. V., Campos, R. H., Martino, V. S., & Cavallaro, L. V. (2013). In vitro antiviral activity of plant extracts from Asteraceae medicinal plants. *Virology Journal, 10*, 245-245. doi:10.1186/1743-422x-10-245

Wang, L., Yang, R., Yuan, B., Liu, Y., & Liu, C. (2015). The antiviral and antimicrobial activities of licorice, a widely-used Chinese herb. *Acta Pharmaceutica Sinica B, 5*(4), 310-315. doi:<http://dx.doi.org/10.1016/j.apsb.2015.05.005>

Wei, B., Cha, S.-Y., Kang, M., Kim, Y. J., Cho, C.-W., Rhee, Y. K., . . . Jang, H.-K. (2015). Antiviral activity of Chongkukjang extracts against influenza A virus in vitro and in vivo. *Journal of Ethnic Foods, 2*(2), 47-51. doi:<https://doi.org/10.1016/j.jef.2015.04.001>

Welch, C. J., Larsson, A., Ericson, A. C., Oberg, B., Datema, R., & Chattopadhyaya, J. (1985). The chemical synthesis and antiviral properties of an acyclovir-phospholipid conjugate. *Acta Chem Scand B, 39*(1), 47-54.

Wu, L. L., Yang, X. B., Huang, Z. M., Liu, H. Z., & Wu, G. X. (2007). In vivo and in vitro antiviral activity of hyperoside extracted from Abelmoschus manihot (L) medik. *Acta Pharmacol Sin, 28*(3), 404-409.

Yang, Z. F., Bai, L. P., Huang, W. B., Li, X. Z., Zhao, S. S., Zhong, N. S., & Jiang, Z. H. (2014). Comparison of in vitro antiviral activity of tea polyphenols against influenza A and B viruses and structure-activity relationship analysis. *Fitoterapia, 93*, 47-53.

Yao, X., Wu, D., Dong, N., Ouyang, P., Pu, J., Hu, Q., . . . Huang, J. (2016). Moracin C, A Phenolic Compound Isolated from Artocarpus heterophyllus, Suppresses Lipopolysaccharide-Activated Inflammatory Responses in Murine Raw264.7 Macrophages. *Int J Mol Sci, 17*(8).

Zeng, P., Zhang, Y., Pan, C., Jia, Q., Guo, F., Li, Y., . . . Chen, K. (2013). Advances in studying of the pharmacological activities and structure–activity relationships of natural C-glycosylflavonoids. *Acta Pharmaceutica Sinica B, 3*(3), 154-162. doi:<https://doi.org/10.1016/j.apsb.2013.04.004>

Zhong, X., Wang, X., Chen, L., Ruan, X., Li, Q., Zhang, J., . . . Xue, W. (2017). Synthesis and biological activity of myricetin derivatives containing 1,3,4-thiadiazole scaffold. *Chem Cent J, 11*(1), 017-0336.
