# Supplementary material for: Computational screening of known broad-spectrum antiviral small organic molecules for potential influenza HA stem inhibitors
Source: PLoS One. 2018 Sep 4;13(9):e0203148. doi: 10.1371/journal.pone.0203148 (PMC6122827; doi:10.1371/journal.pone.0203148)
Supplement: S3 Table — (DOCX) [file pone.0203148.s003.docx]

| **LogP Prediction [Log Units]** | **Ready Biodegradability** | **Developmental Toxicity model** | **Carcinogenicity model** | **Mutagenicity Model (CAESAR)** | **QSAR Models** |
| --- | --- | --- | --- | --- | --- |
| 3.22 | Readily Biodegradable | NON-Toxicant | NON-Carcinogen | NON-Mutagenic | Cucurmin |
| 1.88 | NON Readily Biodegradable | Toxicant | Carcinogen | NON-Mutagenic | Caesalmin B |
| 2.17 | NON Readily Biodegradable | Toxicant | NON-Carcinogen | Mutagenic | Glycyrrhiza Flavonol A |
| 2.94 | NON Readily Biodegradable | Toxicant | NON-Carcinogen | Mutagenic | Griffithdione |
| 2.62 | NON Readily Biodegradable | Toxicant | NON-Carcinogen | NON-Mutagenic | Silybin B |
| 3.25 | NON Readily Biodegradable | NON-Toxicant | Carcinogen | NON-Mutagenic | Spirooliganone A |
| -0.93 | NON Readily Biodegradable | Toxicant | NON-Carcinogen | NON-Mutagenic | EGCG |
| 0.97 | Readily Biodegradable | Toxicant | NON-Carcinogen | NON-Mutagenic | Ladanein |
| 6.22 | Readily Biodegradable | Toxicant | Carcinogen | NON-Mutagenic | Caapsanthin |
| 2.35 | Readily Biodegradable | Toxicant | Carcinogen | NON-Mutagenic | Taraxacin |
| 2.36 | Readily Biodegradable | Toxicant | NON-Carcinogen | NON-Mutagenic | Thujone |
| -2.69 | NON Readily Biodegradable | Toxicant | NON-Carcinogen | NON-Mutagenic | Hesperidin |
| -2.69 | NON Readily Biodegradable | Toxicant | NON-Carcinogen | NON-Mutagenic | Scopoletin |
| -0.03 | Readily Biodegradable | Toxicant | NON-Carcinogen | NON-Mutagenic | Isorhamnetin |
| -0.05 | Readily Biodegradable | Toxicant | NON-Carcinogen | NON-Mutagenic | Polygalaxanthone III |
| 2.4 | NON Readily Biodegradable | Toxicant | NON-Carcinogen | NON-Mutagenic | Sangenon |
| 0.99 | Readily Biodegradable | Toxicant | NON-Carcinogen | NON-Mutagenic | Baicaein |
| 2.52 | NON Readily Biodegradable | Toxicant | Carcinogen | NON-Mutagenic | Naringenin |
| -1.34 | NON Readily Biodegradable | Toxicant | NON-Carcinogen | Mutagenic | Apigenin |
| -0.28 | Readily Biodegradable | Toxicant | NON-Carcinogen | Mutagenic | Quercetin |
| 2.53 | Readily Biodegradable | Toxicant | NON-Carcinogen | NON-Mutagenic | Luteolin |
| 3.88 | Not classifiable | Toxicant | Carcinogen | NON-Mutagenic | Honokiol |
| 0.39 | Readily Biodegradable | Toxicant | NON-Carcinogen | NON-Mutagenic | Nobelitin |
| -5.95 | Readily Biodegradable | Toxicant | NON-Carcinogen | Mutagenic | Punicalagin |
| 2.9 | Readily Biodegradable | Toxicant | Carcinogen | NON-Mutagenic | Isoliquiritgenin |
| 0.41 | NON Readily Biodegradable | Toxicant | NON-Carcinogen | Mutagenic | Homoplantagnin |
| 0.15 | NON Readily Biodegradable | Toxicant | NON-Carcinogen | Mutagenic | Nepitrin |
| -0.59 | Readily Biodegradable | Toxicant | NON-Carcinogen | Mutagenic | 6-hydroxyluteolin 7-O-glycoside |
| -0.28 | Readily Biodegradable | Toxicant | NON-Carcinogen | Mutagenic | 7-O-B-glucopyranoside |
| 0.51 | NON Readily Biodegradable | Toxicant | NON-Carcinogen | NON-Mutagenic | Catechin |
| -0.59 | Readily Biodegradable | Toxicant | NON-Carcinogen | Mutagenic | Hyperoside |
| -0.28 | Readily Biodegradable | Toxicant | NON-Carcinogen | Mutagenic | Quercetin |
| -1.45 | Readily Biodegradable | Toxicant | NON-Carcinogen | NON-Mutagenic | Rutin |
| -2.88 | NON Readily Biodegradable | NON-Toxicant | NON-Carcinogen | Mutagenic | 1,1 dipenyl-2- pcrylhydrazol |
| 7.05 | Readily Biodegradable | NON-Toxicant | NON-Carcinogen | NON-Mutagenic | Linoleic acid |
| -1.99 | NON Readily Biodegradable | NON-Toxicant | NON-Carcinogen | Mutagenic | Castanospermine |
| 2.8 | NON Readily Biodegradable | NON-Toxicant | NON-Carcinogen | NON-Mutagenic | Glycyrrhizin |
| 5.21 | Readily Biodegradable | Toxicant | NON-Carcinogen | Mutagenic | 3-ethyl-4-methypentanol |
| -2.8 | NON Readily Biodegradable | NON-Toxicant | NON-Carcinogen | NON-Mutagenic | D-glucosamine |
| -2.14 | Readily Biodegradable | NON-Toxicant | NON-Carcinogen | NON-Mutagenic | 2-deoxy-D-glucose |
| 3.52 | Readily Biodegradable | Toxicant | Carcinogen | NON-Mutagenic | Chrysin |
| 2.04 | Readily Biodegradable | Toxicant | NON-Carcinogen | NON-Mutagenic | Galangin |
| 1.77 | Readily Biodegradable | Toxicant | NON-Carcinogen | Mutagenic | Kempferol |
| 1.54 | Readily Biodegradable | Toxicant | NON-Carcinogen | Mutagenic | Morin |
| 1.24 | Readily Biodegradable | Toxicant | NON-Carcinogen | Mutagenic | Dinatin |
| -0.06 | NON Readily Biodegradable | Toxicant | NON-Carcinogen | Mutagenic | Vitexin |
| 0.37 | NON Readily Biodegradable | Toxicant | NON-Carcinogen | Mutagenic | Scutellarin |
| 0.16 | NON Readily Biodegradable | Toxicant | NON-Carcinogen | Mutagenic | Galuteolin |
| 2.02 | Readily Biodegradable | Toxicant | NON-Carcinogen | NON-Mutagenic | Rhamnocitrin |
| 0.66 | NON Readily Biodegradable | Toxicant | NON-Carcinogen | Mutagenic | Liquiiritin |
| 1.24 | Readily Biodegradable | Toxicant | NON-Carcinogen | Mutagenic | Myricetin |
| 2.06 | Readily Biodegradable | Toxicant | NON-Carcinogen | NON-Mutagenic | Genistein |
| 2.58 | Readily Biodegradable | Toxicant | Carcinogen | NON-Mutagenic | Formononetin |
| 2.33 | Readily Biodegradable | Toxicant | Carcinogen | NON-Mutagenic | Daidzein |
| -2.69 | NON Readily Biodegradable | Toxicant | NON-Carcinogen | NON-Mutagenic | Hesperidin |
| 2.33 | Readily Biodegradable | NON-Toxicant | Carcinogen | NON-Mutagenic | Sulphuretin |
| 2.4 | NON Readily Biodegradable | Toxicant | NON-Carcinogen | NON-Mutagenic | Sanggenon |
| 5.4 | NON Readily Biodegradable | Toxicant | NON-Carcinogen | NON-Mutagenic | Kuwanon L |
| 3.54 | NON Readily Biodegradable | Toxicant | Carcinogen | NON-Mutagenic | Moracin |
| 1.17 | NON Readily Biodegradable | Toxicant | Carcinogen | Mutagenic | Theaflavin |
| 3.39 | NON Readily Biodegradable | Toxicant | NON-Carcinogen | NON-Mutagenic | Procyanidin |
| 3.14 | NON Readily Biodegradable | Toxicant | NON-Carcinogen | Mutagenic | Flavanone |
| -0.03 | Readily Biodegradable | Toxicant | NON-Carcinogen | NON-Mutagenic | Isorhamnetin |
| 3.41 | NON Readily Biodegradable | Toxicant | Carcinogen | NON-Mutagenic | 7-O-galloytricetiflavon |
| -1.82 | NON Readily Biodegradable | Toxicant | NON-Carcinogen | NON-Mutagenic | Matteflavoside |
| 3.83 | Possible Readily Biodegradable | NON-Toxicant | NON-Carcinogen | NON-Mutagenic | Cinaserin |
| 0.51 | NON Readily Biodegradable | Toxicant | NON-Carcinogen | NON-Mutagenic | Epicatechins |
| 3.41 | NON Readily Biodegradable | Toxicant | Carcinogen | NON-Mutagenic | Thalimonine |
| 1.9 | Readily Biodegradable | Toxicant | Carcinogen | Mutagenic | Coumarins |
| 3.57 | NON Readily Biodegradable | Toxicant | NON-Carcinogen | NON-Mutagenic | Diterpene |
| 0.06 | NON Readily Biodegradable | Toxicant | Carcinogen | Mutagenic | Licorice |
| 4.36 | Readily Biodegradable | Toxicant | Carcinogen | NON-Mutagenic | Triterpene Saponin |
| 2.32 | Readily Biodegradable | Toxicant | Carcinogen | NON-Mutagenic | Biochanin |
| 0.99 | Readily Biodegradable | Toxicant | NON-Carcinogen | NON-Mutagenic | Baicalein |
| 2.32 | Readily Biodegradable | Toxicant | NON-Carcinogen | NON-Mutagenic | Isocutellarein |
| 1.68 | NON Readily Biodegradable | Toxicant | NON-Carcinogen | NON-Mutagenic | Quinocarcin |
| 0.06 | NON Readily Biodegradable | Toxicant | Carcinogen | Mutagenic | Fulvic acid |
| 4.36 | Readily Biodegradable | Toxicant | Carcinogen | NON-Mutagenic | Amentoflavone |
| 4.36 | Readily Biodegradable | Toxicant | Carcinogen | NON-Mutagenic | Robustaflavone |
| 4.36 | Readily Biodegradable | Toxicant | Carcinogen | NON-Mutagenic | Agatisflavone |
| 10.8 | Readily Biodegradable | Toxicant | NON-Carcinogen | NON-Mutagenic | Laurifolin |
| 0.68 | Not classifiable | NON-Toxicant | Carcinogen | NON-Mutagenic | Elatin |
| 3.57 | NON Readily Biodegradable | Toxicant | NON-Carcinogen | NON-Mutagenic | Lanceolatin |
| 4.46 | NON Readily Biodegradable | Toxicant | NON-Carcinogen | NON-Mutagenic | Hispidone |
| 1.99 | Readily Biodegradable | Toxicant | Carcinogen | NON-Mutagenic | Eupatin |
| 4.42 | NON Readily Biodegradable | Toxicant | Carcinogen | NON-Mutagenic, | Glabranin |
| 2.04 | Readily Biodegradable | Toxicant | NON-Carcinogen | NON-Mutagenic | Galangin |
| 2.05 | Readily Biodegradable | Toxicant | Carcinogen | Mutagenic | Ternatin |
| 2.26 | Readily Biodegradable | Toxicant | NON-Carcinogen | NON-Mutagenic | Salicyclic acid |
| 1.32 | NON Readily Biodegradable | Toxicant | NON-Carcinogen | Mutagenic | Efinrin |
| 0.24 | NON Readily Biodegradable | Toxicant | NON-Carcinogen | NON-Mutagenic | Gibbercellin |
| 3.08 | Readily Biodegradable | Toxicant | Carcinogen | Mutagenic | Phosphatidic acid |
| 4.41 | NON Readily Biodegradable | Toxicant | NON-Carcinogen | NON-Mutagenic | Ferutinin |
| -1.22 | NON Readily Biodegradable | NON-Toxicant | NON-Carcinogen | NON-Mutagenic | Salicin |
| -2.72 | Readily Biodegradable | NON-Toxicant | NON-Carcinogen | NON-Mutagenic | Sorbitol |
| 3.08 | Readily Biodegradable | Toxicant | Carcinogen | Mutagenic | Graveoline |
| -1.76 | Readily Biodegradable | NON-Toxicant | NON-Carcinogen | NON-Mutagenic | Ascorbic acid |
| 3.08 | Readily Biodegradable | Toxicant | Carcinogen | Mutagenic | Pendulinin |
| 0.36 | Not classifiable | NON-Toxicant | NON-Carcinogen | NON-Mutagenic | Nicotinic acid |
