# Supplementary material for: Computational screening of known broad-spectrum antiviral small organic molecules for potential influenza HA stem inhibitors
Source: PLoS One. 2018 Sep 4;13(9):e0203148. doi: 10.1371/journal.pone.0203148 (PMC6122827; doi:10.1371/journal.pone.0203148)
Supplement: S4 Table — (DOCX) [file pone.0203148.s004.docx]

| Docking energy  (Kcal/mol) | Residue Interactions | Number of HBonds | Hbond Energy (Kcal/mol) | Interaction Energy  (Kcal/mol) | Derivative Name | S.No. |
| --- | --- | --- | --- | --- | --- | --- |
| -15.62 | HIS28, THR92,ASN97,THR100,VAL103,ILE107 | 6 | -2.356 | -7.36 | Silybin |  |
| -27.32 | LEU32,GLY71,TRP72,GLN93,ILE96,VAL103 | 5 | -3.214 | -6.36 | Apigenin |  |
| -19.66 | GLN93,ILE96,THR100,ASN104 | 4 | -4.522 | -7.52 | Morin |  |
| -18.65 | GLY71,THR92,ILE96,VAL103 | 4 | -4.588 | -8.69 | Homoplantagnin |  |
| -28.74 | ASN31, LEU32,THR100 | 3 | -2.496 | -5.66 | Naringenin |  |
| -16.68 | TRP72,GLN93,ILE96,ASN97 | 4 | -3.215 | -5.87 | Epicatechin |  |
| -11.66 | THR92,ASN97,ASN104,ILE107 | 4 | -3.754 | -6.88 | Sorbitol |  |
| -16.66 | VAL30,ASP70,THR100 | 3 | -6.512 | -4.95 | Glycyrrhiza Flavonol A |  |
| -10.62 | GLN93,THR100,ASN104 | 3 | -2.545 | -4.21 | Hesperidin |  |
| -25.68 | ASN31,ASN97,THR100 | 3 | -2.634 | -3.22 | Spirooligannone |  |
| -14.27 | HIS28,GLN93,THR100 | 3 | -3.544 | -6.95 | EGCG |  |
| -16.85 | TRP72,GLN93,ILE96 | 3 | -5.142 | -2.58 | Griffithdione |  |
| -16.89 | HIS28,ASP70.THR100 | 3 | -4.561 | -2.99 | Galangin |  |
| -11.86 | GLY71,TRP72,ASN104 | 3 | -3.653 | -4.55 | Ternatin |  |
| -19.38 | LEU32,TRP72,GLN93 | 3 | -6.356 | -3.23 | Lanceolatin |  |
| -9.86 | THR92,GLN93,THR100 | 3 | -3.544 | -3.21 | Diterpene |  |
| -22.15 | VAL30,GLN93,THR100 | 3 | -6.154 | -6. 32 | Fulvic acid |  |
| -7.85 | THR92,ASN104,ILE107 | 3 | -8.356 | -5.55 | Robustaflavone |  |
| -12.55 | ASN31,TRP72,ILE107 | 3 | -5.654 | -4.35 | Nepitrin |  |
| -13.01 | ASN31,TRP72,THR100 | 3 | -3.444 | -5.21 | Kuwanon L |  |
| -16.55 | THR92,GLN93,ASN97 | 3 | -1.344 | -5.01 | Catechin |  |
| -13.46 | HIS28,VAL103,THR100 | 3 | -8.366 | -5.11 | Baicalein |  |
| -23.63 | ASP70,TRP72,ILE96,VAL103 | 4 | -9.544 | -6.36 | Procyanidin |  |
| -16.85 | ASN31,ASP70,THR92,ILE96 | 4 | -2.574 | -1.53 | Quercetin |  |
| -21.36 | THR92,GLN93,ASN97,ILE107 | 4 | -2.111 | -1.21 | Luteolin |  |
| -18.65 | THR92,ILE96,ASN104,THR100 | 4 | -3.577 | -2.32 | Honokiol |  |
| -19.65 | ASN104,ILE107,THR100 | 3 | -6.633 | -2.36 | Nobelitin |  |
| -19.64 | HIS28,ASP70,THR92 | 3 | -8.652 | -2.22 | Isoliquintgenin |  |
| -17.60 | TP72,ILE96,VAL103 | 3 | -1.532 | -1.22 | Salicyclic acid |  |
| -13.85 | LEU32,TRP72,ASN97 | 3 | -3.545 | -1.85 | Salicin |  |
| -16.32 | GLN93,VAL103,ASN104 | 3 | -6.354 | -2.86 | 7-O-Galloytricetiflavone |  |
| -17.62 | GLN93,ASN97,ASN104 | 3 | -5.645 | 1.87 | amentoflavone |  |
| -15.55 | LEU32,GLN93,THR100 | 3 | -3.214 | 2.36 | Scutellarin |  |
| -16.24 | HIS28,VAL30,ASN104 | 3 | -5.623 | -4.52 | Isorhamnetin |  |
| -16.23 | ASP60,TRP72,ASN97 | 3 | -3.266 | 2.63 | Baicalein |  |
| -11.20 | THR92,ASN97,ILE107 | 3 | -7.833 | -5.38 | Thujone |  |
| -10.09 | GLN93,ASN97,ILE107 | 3 | -3.613 | -2.75 | Ladanein |  |
| -13.68 | VAL30,ASP70,THR100 | 3 | -5.616 | 3.32 | Theoflavin |  |
| -12.20 | ASN31,ASP70,ILE96 | 3 | -8.616 | 4.25 | Matteflavoside |  |
| -18.63 | VAL30,TRP72,ASN97 | 3 | -3.365 | -2.22 | Diterpene |  |
| -18.52 | GLY71, ILE96,ASN104 | 3 | -3.613 | -4.13 | Triterpene |  |
| -16.24 | GLN93,ASN104,ILE107 | 3 | -5.362 | -5.21 | Lanceolatin |  |
| 15.26 | ASN31,ASP70,TRP72 | 3 | -6.315 | -6.25 | Laurifolin |  |
| 11.55 | GLN93,ASN97,VAL103 | 3 | -5.622 | -2.89 | Ferutinin |  |
| 10.54 | TRP72,ILE96,ASN97 | 3 | -8.621 | -3.75 | Nicotinic acid |  |
| 16.78 | HIS28,ASN97,THR100 | 3 | -5.663 | -4.97 | Phosphatidic acid |  |
