# Supplementary material for: Computational screening of known broad-spectrum antiviral small organic molecules for potential influenza HA stem inhibitors
Source: PLoS One. 2018 Sep 4;13(9):e0203148. doi: 10.1371/journal.pone.0203148 (PMC6122827; doi:10.1371/journal.pone.0203148)
Supplement: S5 Table — (DOCX) [file pone.0203148.s005.docx]

| **Residue Interactions** | | **Docking Energy** | **Interaction Energy (Kcal/mol)** | **Hbonds** | **Residue Interactions** | | **Docking Energy** | **Interaction Energy (Kcal/mol)** | **Hbonds** | **Derivative Name** | **S.No.** |
| --- | --- | --- | --- | --- | --- | --- | --- | --- | --- | --- | --- |
|  | **H5** | | | |  | **H2** | | | |  |  |
| HIS38,GLY20,GLN20,HIS18,TYR102,ILE45 | | -20.41 | -7.66 | 6 | TRP21, THR41, PHE45, ASP46THR291, LEU292, ILE42, LYS40, THR318 | | -22.67 | -5.62 | 9 | Procyanidin |  |
| GLY20, HIS18, ASP19, TYR102, GLN42, ILE45, MET54, | | -16.35 | -7.36 | 7 | GLY20, LYS38, TRP21, THR41, PHE45, ASP46, LYS43, GLN42, | | -20.63 | -5.98 | 8 | EGCG |  |
| HIS38, PHE55, GLY20, HIS18, ASP19, TYR102, GLN42, ILE45 | | -12.45 | -7.22 | 8 | VAL18, ASP19, GLY20, LYS38, TRP21, PRO293, THR291, LEU292 | | -20.11 | -7.54 | 8 | Sorbitol |  |
| GLY20, HIS18, ASP19, TYR102, GLN42, | | -10.43 | -8.66 | 5 | ASP46, LYS43, GLN42, HIS38, VAL52 | | -16.28 | -6.32 | 5 | Kuwanon L |  |
| HIS18, ASP19, TYR102, GLN42, ILE45, MET54 | | -11.36 | -3.52 | 6 | HIS38, VAL52, ILE56, PRO293, ILE42, LYS40, THR318, GLY20, | | -17.13 | -5.85 | 8 | Morin |  |
| ASP19, TYR102, GLN42, ILE45, MET54, PHE55, | | -13.42 | -4.66 | 6 | TRP21, THR41, PHE45, ASP46, LYS43, GLN42, | | -15.16 | -2.22 | 6 | Scutellarin |  |
| HIS18, ASP19, TYR102, | | -14.29 | -6.51 | 3 | GLY20, LYS38, THR291, LEU292, ILE42, | | -16.36 | -6.36 | 5 | Hesperidin |  |
| PHE55, GLY20, HIS18 | | -13.29 | -3.95 | 3 | LYS38, TRP21, THR41, PHE45, ASP46, ILE42, LYS40 | | -11.67 | -5.52 | 7 | Epicatechin |  |
| ASP19, ILE45, MET54, PHE55, | | -12.65 | -2.66 | 4 | LYS43, GLN42, HIS38, VAL52, ILE56, | | -13.45 | -6.53 | 5 | Glycyrrhiza Flavonol A |  |
| TYR102, GLN42, ILE45, MET54, PHE55, THR41 | | -9.89 | -3.95 | 6 | GLN42, HIS38, VAL52 | | -14.36 | -5.22 | 3 | Silybin |  |
| HIS18, ASP19, THR41 | | -10.41 | -7.68 | 3 | GLY20, LYS38, TRP21, THR41, PHE45, ASP46, | | -16.25 | -8.56 | 6 | Spirooligannone |  |
| HIS18, ASP19, TYR102, GLN42, ILE45, MET54, | | -13.10 | -2.66 | 6 | PRO293, THR291, LEU292, ILE42, LYS40, THR318 | | -9.66 | -5.62 | 5 | Isorhamnetin |  |
| GLY20, HIS18, ASP19, PHE55, THR41 | | -11.02 | -2.84 | 5 | LYS38, TRP21, THR41, PHE45, ASP46, | | -8.76 | -6.56 | 5 | Baicalein |  |
| GLY20, ILE45, MET54, | | -10.13 | -3.56 | 3 | LYS43, GLN42, GLY20, LYS38, TRP21, THR41, PHE45, | | -11.31 | -4.56 | 7 | Naringenin |  |
| TYR102, GLN42, ILE45, MET54, PHE55, THR41 | | -9.63 | -5.56 | 6 | LYS43, GLN42, LEU292, ILE42, LYS40 | | -12.11 | -6.55 | 5 | Quercetin |  |
| GLY20, HIS18, ASP19, TYR102, | | -7.35 | -2.87 | 4 | TRP21, THR41, PHE45, ASP46, LYS43, | | -13.09 | -8.65 | 5 | Luteolin |  |
| HIS38, PHE55, GLY20 | | -10.31 | -2.99 | 3 | GLY20, LYS38, ILE42, LYS40, | | -10.03 | -9.65 | 4 | Honokiol |  |
| ILE45, MET54, PHE55 | | -9.68 | -6.96 | 3 | ASP19, GLY20, LYS38, TRP21 | | -11.51 | -8.55 | 4 | Apigenin |  |
| GLN42, ILE45, MET54, PHE55, THR41 | | -11.31 | -3.66 | 5 | GLN42, HIS38, VAL52 | | -9.37 | -7.85 | 3 | Isoliquintgenin |  |
| HIS18, ASP19, THR41 | | -6.49 | -2.83 | 3 | GLY20, LYS38, TRP21, THR41, PHE45, | | -8.41 | -4.21 | 5 | Salicyclic acid |  |
| TYR102, GLN42, ILE45, MET54, PHE55, THR41 | | -7.93 | -5.65 | 7 | GLN42, HIS38, VAL52, ILE56, PRO293, THR291, | | -9.36 | -4.32 | 6 | Salicin |  |
| PHE55, GLY20, HIS18, ASP19, TYR102, GLN42, ILE45 | | -9.41 | -2.64 | 7 | GLN42, HIS38, VAL52, ILE56, PRO293, THR291, | | -9.46 | -6.87 | 6 | 7-O-Galloytricetiflavone |  |
| HIS38, PHE55, GLY20, HIS18, ASP19, TYR102 | | -8.89 | -3.69 | 6 | GLY20, LYS38, TRP21, THR41, ILE42, LYS40, THR318 | | -10.30 | -8.69 | 7 | amentoflavone |  |
