# Supplementary material for: Computational screening of known broad-spectrum antiviral small organic molecules for potential influenza HA stem inhibitors
Source: PLoS One. 2018 Sep 4;13(9):e0203148. doi: 10.1371/journal.pone.0203148 (PMC6122827; doi:10.1371/journal.pone.0203148)
Supplement: S6 Table — (DOCX) [file pone.0203148.s006.docx]

| **Residue Interactions** | | **Docking Energy**  **(Kcal/mol)** | **Interaction Energy (Kcal/mol)** | **Hbonds** | **Residue Interactions** | | | **Docking energy**  **(Kcal/mol)** | | **Interaction Energy (Kcal/mol)** | **Hbonds** | **Derivative Name** | **S.No** |  |  |
| --- | --- | --- | --- | --- | --- | --- | --- | --- | --- | --- | --- | --- | --- | --- | --- |
|  | **H7** | | | | |  |  | | **H3** | | | | | |  |
| ALA36, LYS38, GLY20, , GLN42, ASP46, | | -16.46 | -6.55 | 5 | GLY16, VAL18, ASP19, ALA36, ARG25 | | | -16.31 | | -6.66 | 5 | Morin | 1. 1 |  |  |
| THR49, SER40, LEU52, ILE56, ALA292 | | -17.39 | -11.66 | 5 | VAL18, ASP19, LEU318, GLU325, GLN34, ALA35 | | | -14.32 | | -2.22 | 6 | Scutellarin |  |  |  |
| ASP19, ALA36, LYS38, VAL293, ILE45, THR49, | | -16.23 | -17.61 | 6 | GLY16, VAL18, ASP19, LEU318, GLU325, GLN34 | | | -11.45 | | -3.24 | 5 | Apigenin |  |  |  |
| LYS38, GLY20, TRP21, THR41, ILE45, | | -14.43 | -8.66 | 5 | ASP19, LEU318, GLU325, GLN34, ALA35 | | | -9.99 | | -3.32 | 5 | Kuwanon L |  |  |  |
| GLN42, ASP46, THR49, SER40, ALA292, ARG291, VAL293 | | -19.65 | -2.66 | 7 | GLN34, ALA35, ALA36, ARG25, GLY33, THR32, ASN146, GLY150, GLU30 | | | -12.45 | | -5.62 | 9 | Procyanidin |  |  |  |
| GLN42, ASP46, THR49, SER40, LEU52 | | -12.43 | -3.95 | 5 | GLY16, VAL18, ALA35, ALA36, ARG25, GLY33, THR32 | | | -13.46 | | -5.52 | 7 | Epicatechin |  |  |  |
| LYS38, GLY20, TRP21, THR41, ILE45 | | -13.21 | -4.85 | 5 | ALA36, ARG25, GLY33, THR32, ASN146, GLY150, GLU30 | | | -12.25 | | -3.55 | 7 | EGCG |  |  |  |
| ASP19, SER40, LEU52, ILE56, ALA292, | | -14.61 | -4.62 | 5 | ASP19, LEU318, GLU325, GLN34, ALA35, | | | -17.21 | | -2.22 | 5 | Baicalein |  |  |  |
| ALA36,GLN42, ASP46 | | -19.63 | -4.95 | 3 | GLY16, VAL18, ASP19, ARG25, GLY33, THR32, ASN146, | | | -16.49 | | -1.63 | 7 | Naringenin |  |  |  |
| TRP21, THR41, ILE45, GLN42, ASP46 | | -14.35 | -4.36 | 5 | ASN146, GLY150, GLU30, ALA36, ARG25 | | | -15.34 | | -4.62 | 5 | Quercetin |  |  |  |
| GLN42, ASP46, ALA292, ARG291, VAL293, ILE45 | | -13.45 | -2.84 | 6 | ASP19, LEU318, GLU325, GLN34, THR32, ASN146 | | | -14.36 | | -3.21 | 6 | Luteolin |  |  |  |
| LYS38, GLY20, TRP21, THR41, ILE45, GLN42 | | -16.33 | -2.84 | 6 | GLU30, VAL18, ASP19, ALA35, ALA36, ARG25 | | | -10.98 | | -6.56 | 6 | Isoliquintgenin |  |  |  |
| ASP46, THR49, LEU52, ILE56 | | -12.43 | -3.56 | 4 | GLU15, GLY16,THR32, ASN146 | | | -14.45 | | -4.56 | 4 | Salicyclic acid |  |  |  |
| GLN42, ASP46, ARG291, VAL293 | | -12.40 | -5.56 | 4 | VAL18, ASP19, LEU318, GLU325, GLN34, | | | -13.43 | | -6.55 | 5 | Salicin |  |  |  |
| GLY20, THR41, ILE45, GLN42, ASP46, ILE56, ALA292 | | -11.36 | -2.87 | 7 | ALA35, ALA36, ARG25, VAL18, ASP19, GLY33, THR32 | | | -11.45 | | -8.65 | 7 | 7-O-Galloytricetiflavone |  |  |  |
| ASP46, THR49, SER40 | | -8.49 | -1.51 | 3 | ARG25, GLY33, THR32, ASN146, GLY150, GLU30 | | | -7.45 | | -1.24 | 6 | Isorhamnetin |  |  |  |
| LYS38, GLY20, TRP21, THR41, LEU52, ILE56, ALA292 | | -20.43 | -3.86 | 7 | VAL18, ASP19, LEU318, GLU325, GLN34, ALA35, ALA36, ARG25, GLY33, | | | -24.45 | | -6.21 | 9 | Sorbitol |  |  |  |
| GLY20, TRP21, THR41, ILE45, GLN42, | | -16.71 | -5.63 | 5 | ASN146, GLY150, GLY16, VAL18, ASP19 | | | -13.69 | | -3.22 | 5 | Amentoflavone |  |  |  |
