# Supplementary material for: Computational screening of known broad-spectrum antiviral small organic molecules for potential influenza HA stem inhibitors
Source: PLoS One. 2018 Sep 4;13(9):e0203148. doi: 10.1371/journal.pone.0203148 (PMC6122827; doi:10.1371/journal.pone.0203148)
Supplement: S7 Table — (DOCX) [file pone.0203148.s007.docx]

| **S.No** | **Compounds** | **Molecular formula** | **Docking Energy**  **(Kcal/mol)** | **Interaction energy**  **(Kcal/mol)** | **Hbond** |
| --- | --- | --- | --- | --- | --- |
| 1 | SID:103512718 | \| 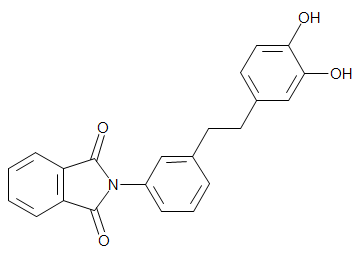 \|  \| \| --- \| --- \| | -8.97 | -3.45 | 5 |
| 2 | SID:160699960 | 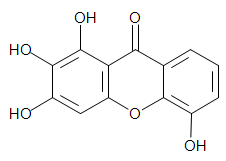 | -9.76 | -1.34 | 4 |
| 3 | SID:103619997 | 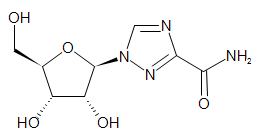 | -7.90 | -1.11 | 4 |
| 4 | SID:103217472 | 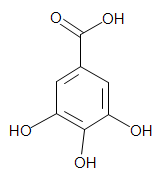 | -7.45 | -2.33 | 4 |
| 5 | SID:242620267 | 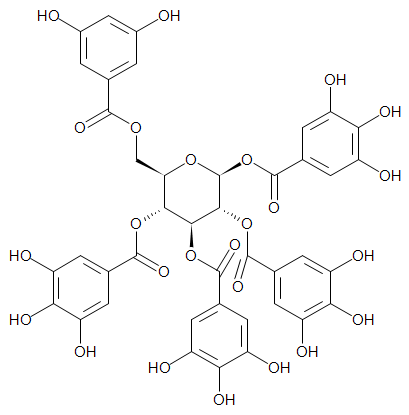 | -8.98 | -1.78 | 4 |
| 6 | SID:242620266 | 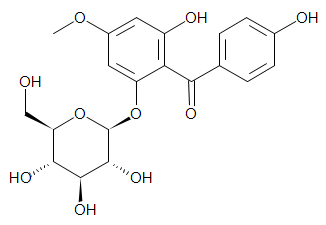 | -8.35 | -1.84 | 4 |
| 7 | SID:242620268 | 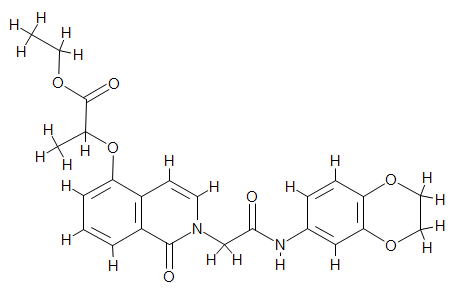 | -8.64 | -2.43 | 4 |
| 8 | SID:163315261 | 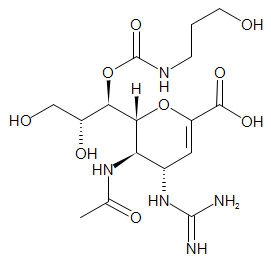 | -7.34 | -1.93 | 3 |
| 9 | SID:163322216 | 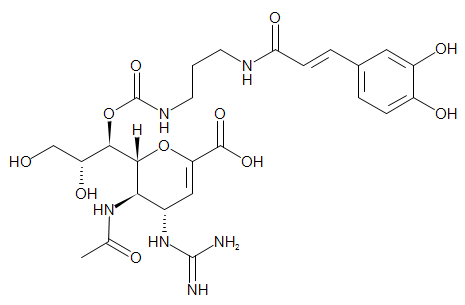 | -6.98 | -1.98 | 3 |
| 10 | SID:160684110 | 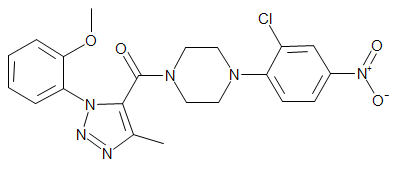 | -6.49 | -3.02 | 3 |
