# Supplementary figures and images for: Computational screening of known broad-spectrum antiviral small organic molecules for potential influenza HA stem inhibitors
Source: PLoS One. 2018 Sep 4;13(9):e0203148. doi: 10.1371/journal.pone.0203148 (PMC6122827; doi:10.1371/journal.pone.0203148)

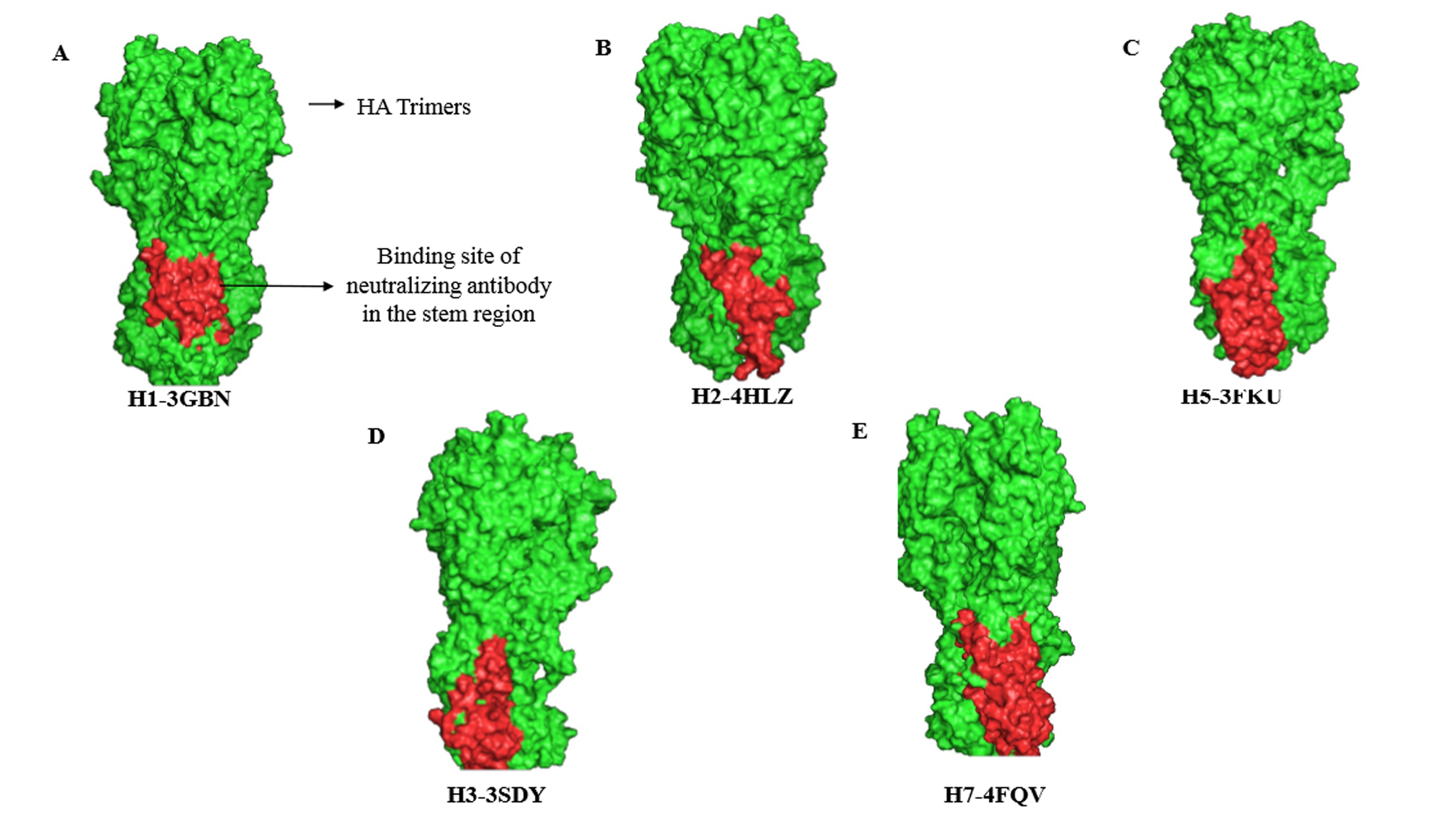

Supplement: S1 Fig — The HA trimers are colored in green with epitopes targeted by bnAb colored dark brown. (TIF) [file pone.0203148.s008.tif]

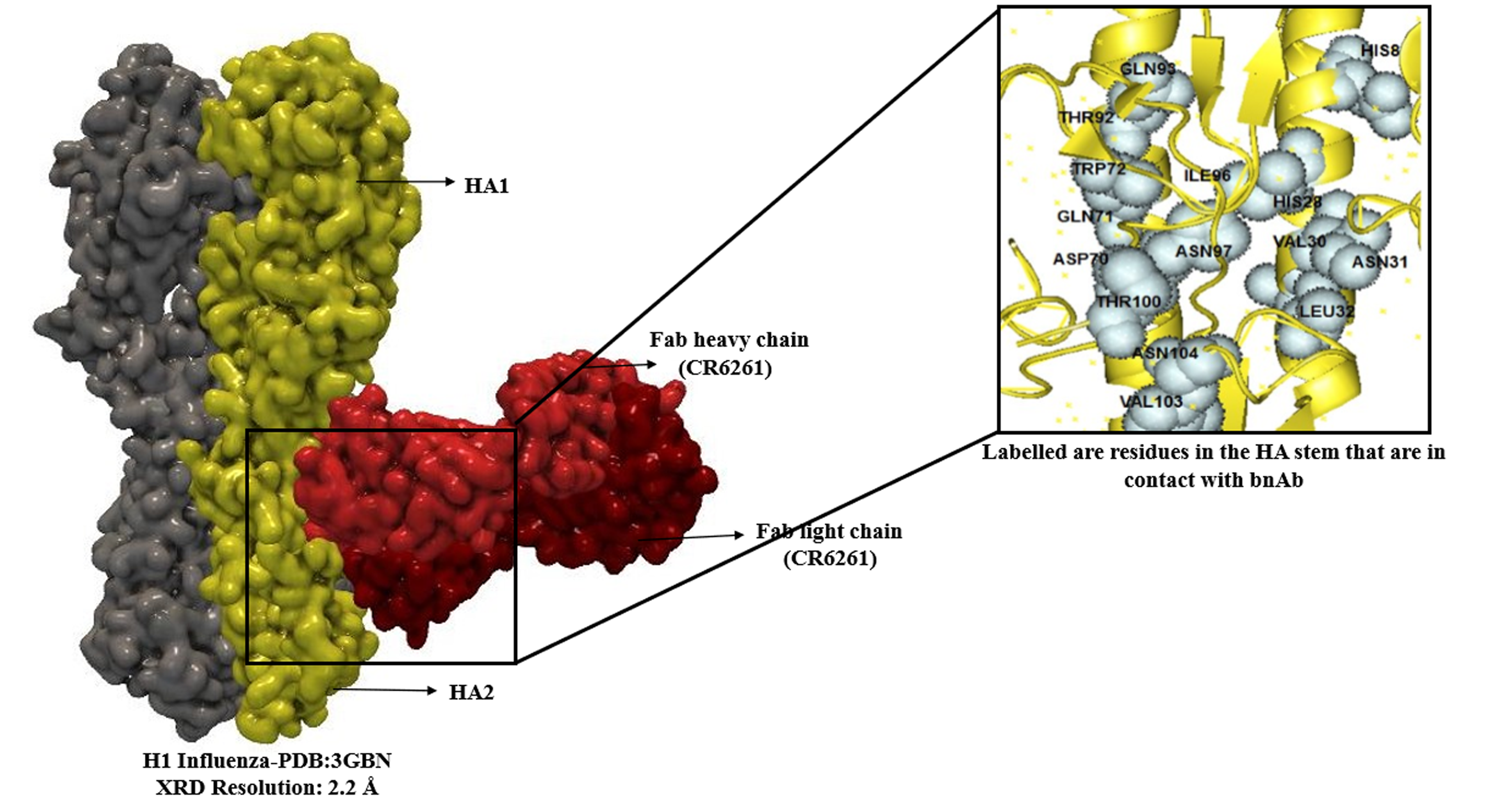

Supplement: S2 Fig — Fab heavy and light chains and HA trimer are depicted in surface representation. Contact residues in the bNAb epitope are labeled in the right panel. (TIF) [file pone.0203148.s009.tif]

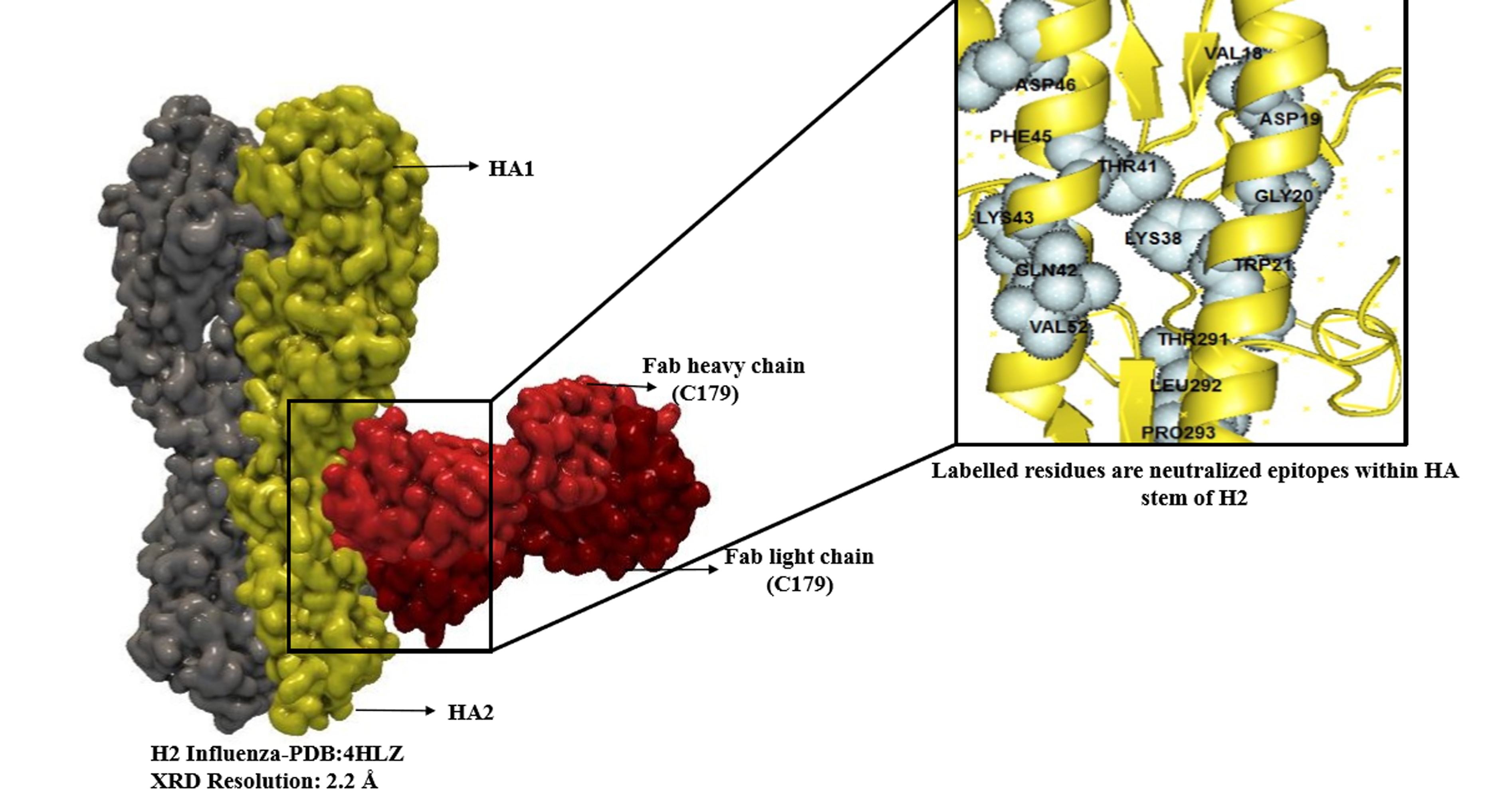

Supplement: S3 Fig — Fab heavy and light chains and HA trimer are depicted in surface representation. Contact residues in the bNAb epitope are labeled in the right panel. (TIF) [file pone.0203148.s010.tif]

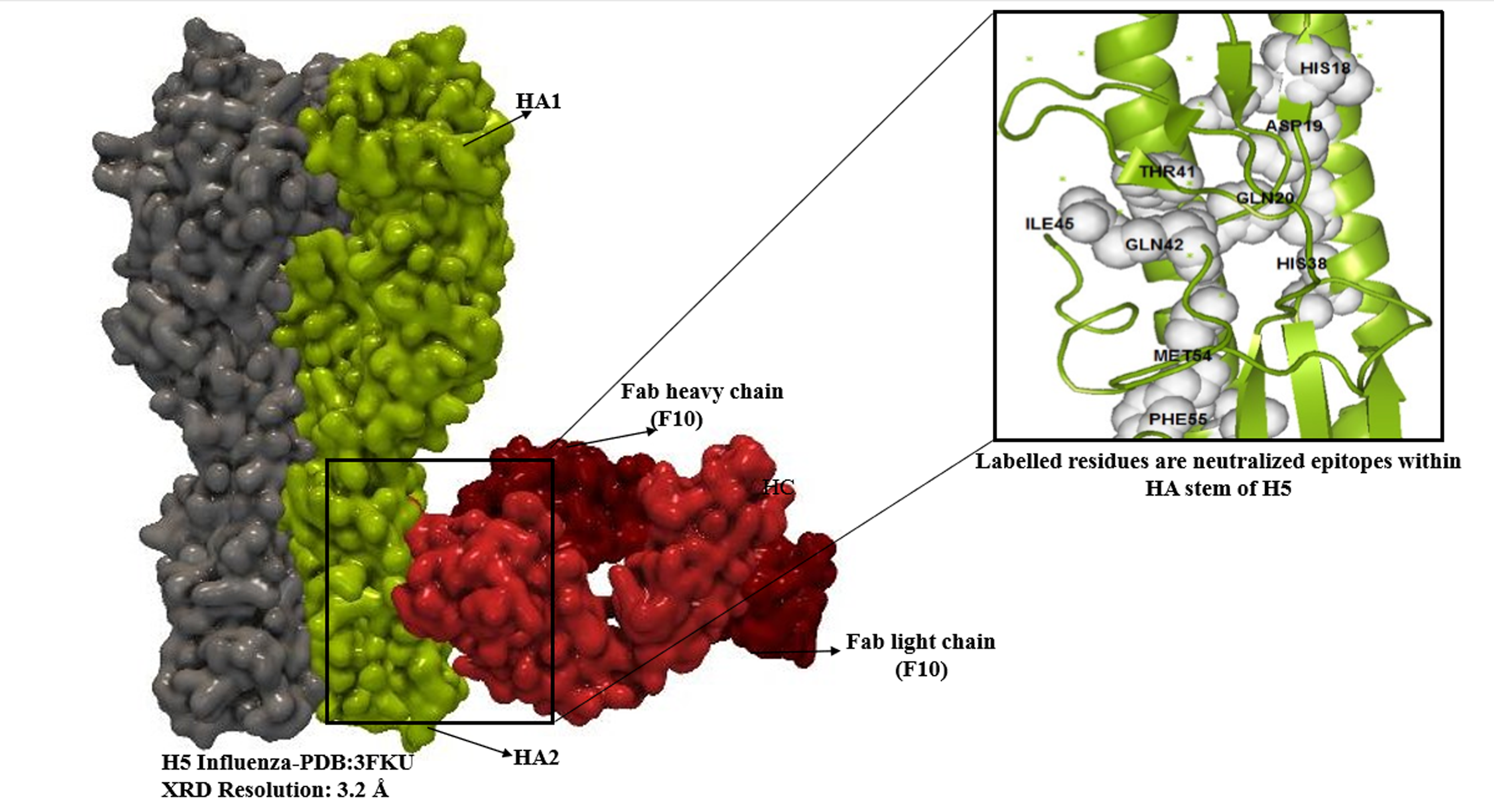

Supplement: S4 Fig — Fab heavy and light chains and HA trimer are depicted in surface representation. Contact residues in the bNAb epitope are labeled in the right panel. (TIF) [file pone.0203148.s011.tif]

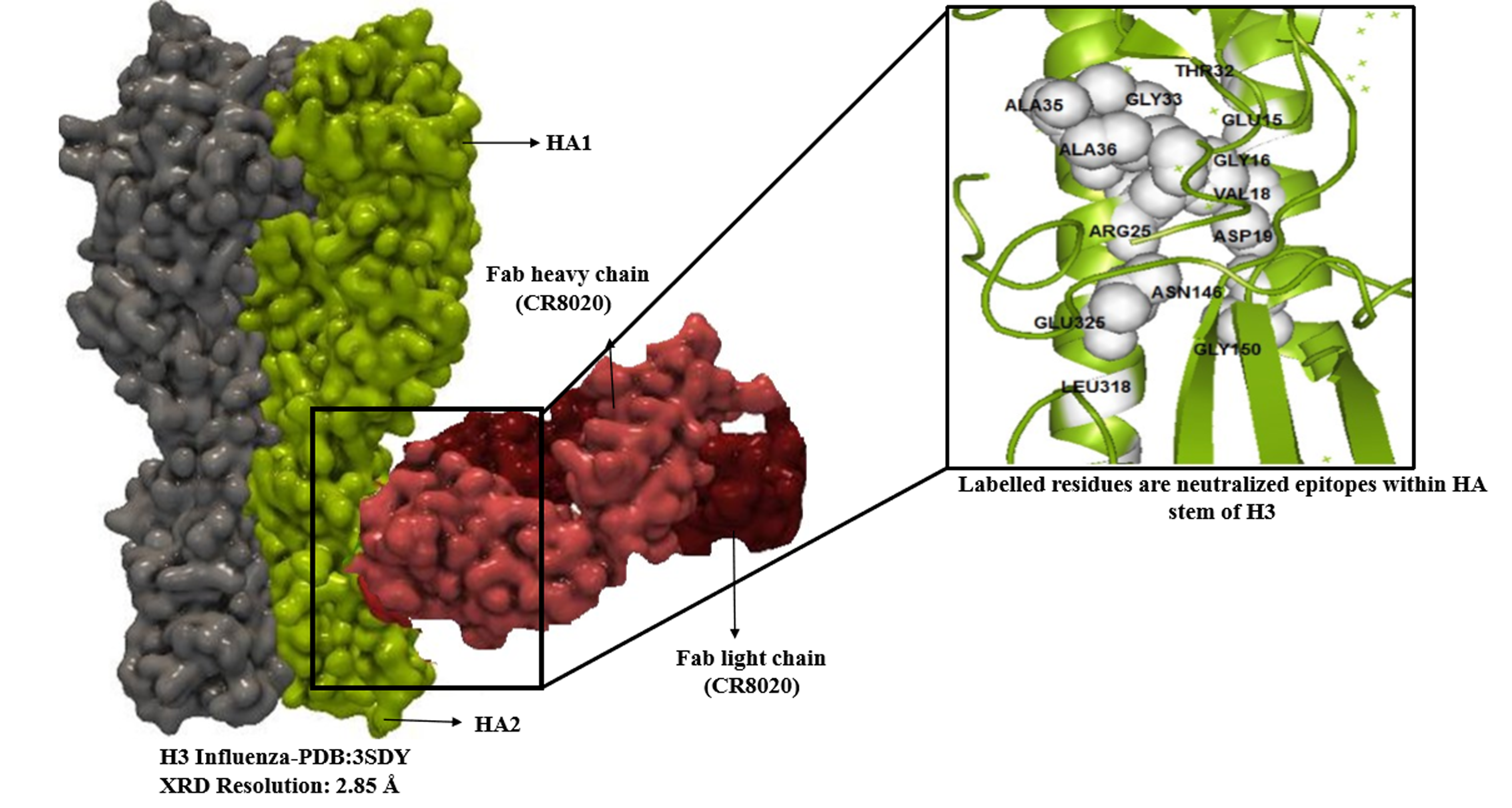

Supplement: S5 Fig — Fab heavy and light chains and HA trimer are depicted in surface representation. Contact residues in the bNAb epitope are labeled in the right panel. (TIF) [file pone.0203148.s012.tif]

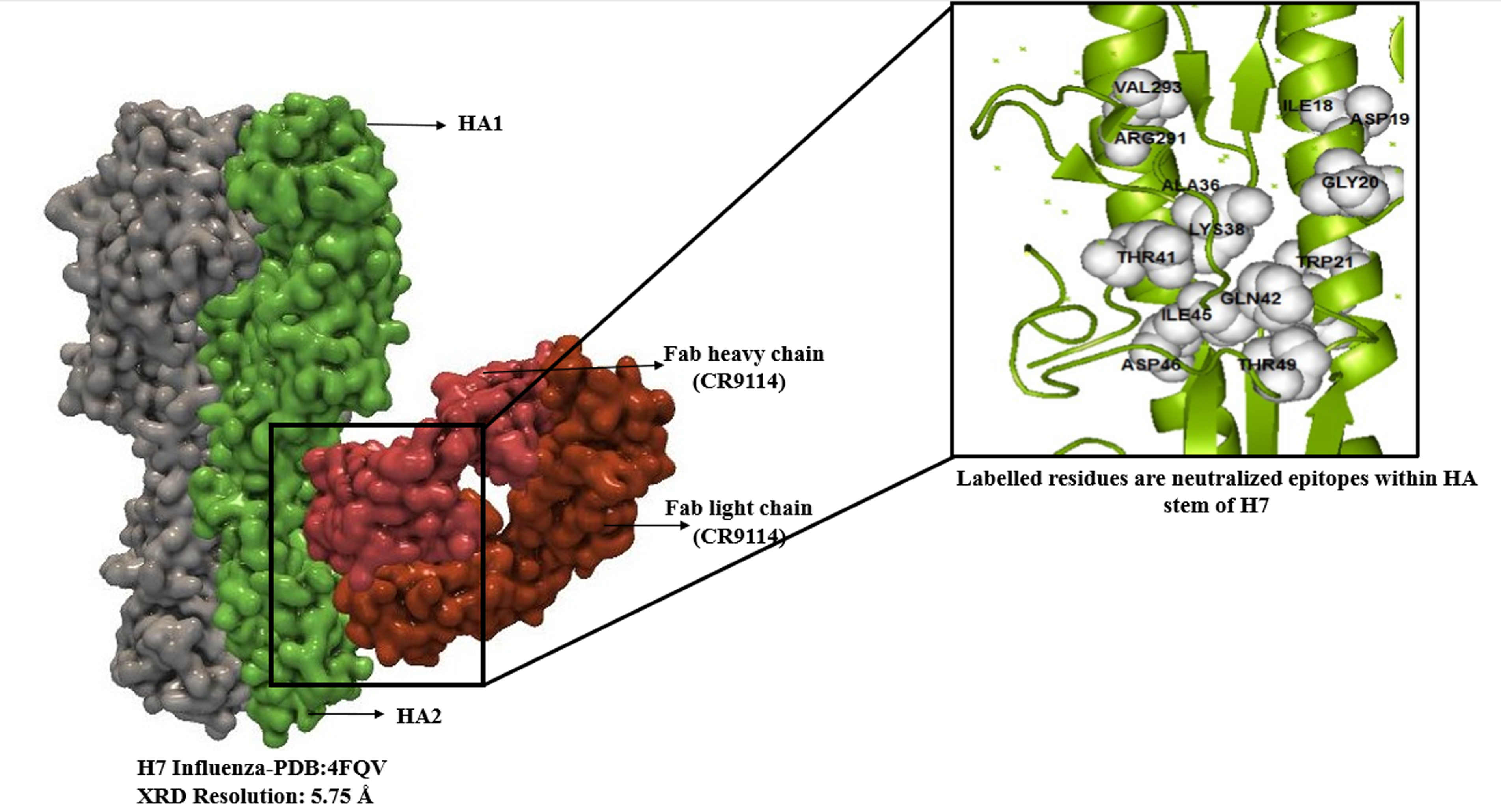

Supplement: S6 Fig — Fab heavy and light chains and HA trimer are depicted in surface representation. Contact residues in the bNAb epitope are labeled in the right panel. (TIF) [file pone.0203148.s013.tif]
